# BRAF V600E and *Pten* deletion in mice produces a histiocytic disorder with features of Langerhans cell histiocytosis

**DOI:** 10.1371/journal.pone.0222400

**Published:** 2019-09-17

**Authors:** David S. Nelson, Ryan L. Marano, Yechaan Joo, Sara Y. Tian, Bhumi Patel, Daniel H. Kaplan, Mark J. Shlomchik, Kristen Stevenson, Roderick T. Bronson, Barrett J. Rollins

**Affiliations:** 1 Department of Medical Oncology, Dana-Farber Cancer Institute, Harvard Medical School, Boston, MA, United States of America; 2 Department of Immunology, University of Pittsburgh School of Medicine, Pittsburgh, PA, United States of America; 3 Department of Dermatology, University of Pittsburgh School of Medicine, Pittsburgh, PA, United States of America; 4 Department of Biostatistics and Computational Biology, Dana-Farber Cancer Institute, Boston, MA, United States of America; 5 Department of Microbiology and Immunobiology, Harvard Medical School, Boston, MA, United States of America; 6 Department of Medicine, Brigham & Women's Hospital and Harvard Medical School, Boston, MA, United States of America; Florida International University, UNITED STATES

## Abstract

Langerhans cell histiocytosis (LCH) is characterized by the accumulation of Langerin (CD207)-expressing histiocytes. Mutational activation of mitogen-activated protein kinase pathway genes, in particular *BRAF*, drives most cases. To test whether activated BRAF is sufficient for the development of LCH, we engineered mice to express BRAF V600E under the control of the human *Langerin* promoter. These mice have shortened survivals, smaller lymphoid organs, absent Leydig cells, and fewer epidermal LCs than controls, but do not accumulate histiocytes. To test whether the absence of histiocyte proliferation could be due to oncogene-induced senescence, we engineered homozygous *Pten* loss in the same cells that expressed BRAF V600E. Like mice with intact *Pten*, these mice have shortened survivals, smaller thymi, and absent Leydig cells. However, loss of *Pten* also leads to the accumulation of CD207^+^ histiocytes in spleen, thymus, and some lymph nodes. While many CD207^+^ histiocytes in the thymus are CD8^-^, reminiscent of LCH cells, the CD207^+^ histiocytes in the spleen and lymph nodes are CD8^+^. These mice also accumulate large numbers of CD207^-^ cells in the lamina propria (LP) of the small intestine. Both the lymphoid and LP phenotypes are likely due to human Langerin promoter-driven BRAF V600E expression in resident CD8^+^ dendritic cells in the former and LP dendritic cells in the latter and confirm that *Pten* loss is required to overcome inhibitory pathways induced by BRAF V600E expression. The complex phenotype of these mice is a consequence of the multiple murine cell types in which the human *Langerin* promoter is active.

## Introduction

Langerhans cell histiocytosis (LCH) is a rare disease characterized by the accumulation of histiocytes having features reminiscent of Langerhans cells [1]. Although predominantly a disease of childhood, LCH can occur at any age and has a broad spectrum of clinical behaviors ranging from a mild, self-limited disease to an aggressive multi-system disorder with significant mortality. In all cases examined to date, the abnormal LCH histiocytes have evidence of constitutive activation of the mitogen-activated protein kinase (MAPK) pathway caused, most often, by somatic activating mutations in genes encoding components of this pathway: *BRAF* mutations in 50% (mostly encoding the BRAF V600E variant), *MAP2K1* mutations in an additional 25%, and a variety of mutations or rearrangements in these or other genes accounting for some, but not all, of the remainder [2–5]. The essential “driver” role for these abnormalities in LCH has been proven by the remarkable clinical responses seen in patients with *BRAF* or *MAP2K1* mutations who are treated with RAF or MEK inhibitors [6–8].

While these observations have advanced our understanding of LCH and provided new therapeutic targets, they have also led to new questions. For example, in other neoplastic diseases driven by activated BRAF, such as melanoma, expression of this strong oncogene in normal precursor cells leads to oncogene-induced senescence, presumed to be an organism-level protective response to oncogenic transformation [9]. Development of cancer in that setting requires disabling of the genes responsible for the senescence response such as *TP53* or *PTEN* [10]. Concomitant mutations in these genes along with *BRAF* in melanoma and other cancers provide evidence for this mechanism [10, 11]. However, LCH samples with mutations that activate the MAP kinase pathway only rarely have additional mutations in genes that drive other pathways. This observation suggests two alternative possibilities: either LCH precursor cells are uniquely able to accommodate a powerful dominant oncogene and respond by proliferating or, like other cells, they also require inactivation of a senescence pathway which has not yet been identified. The lack of LCH precursor cell lines makes this a particularly daunting question to answer.

Another unanswered question concerns LCH’s cell of origin. Although the abnormal histiocytes in LCH share many features with mature Langerhans cells, including expression of CD1a and CD207/Langerin, mRNA expression patterns of LCH cells are more similar to myeloid precursor cells than mature Langerhans cells [12]. In addition, patients who have multi-system LCH with *BRAF* mutations in their LCH cells also have *BRAF* mutations in their hematopoietic stem cell populations [13]. This has led to a compelling hypothesis that the cell of origin for LCH is a hematopoietic precursor and that the clinical manifestations of LCH depend on where along the differentiation pathway the oncogenic *BRAF* mutation occurs [1]. Again, however, the absence of cell lines corresponding to LCH precursors has confounded attempts to test this hypothesis mechanistically.

We have attempted to approach these questions using *in vivo* modeling. We have generated mice expressing BRAF V600E under the control of the human Langerin promoter to determine if this dominant oncogene is sufficient to cause accumulation of LCH-like cells. Previous work from others shows that this is the case when the murine Langerin promoter drives BRAF V600E expression [13]. In contrast, our model, which uses the human Langerin promoter, does not feature histiocyte accumulation. To address the question of whether a second genetic alteration is required, we engineered biallelic inactivation of *Pten* in the cells that express BRAF V600E. These mice develop a complex proliferative disorder with aspects that are reminiscent of LCH. Together, these results provide insight into mechanisms of histiocyte transformation which may be relevant to LCH.

## Materials and methods

### Mice

*Braf*^*CA*^*Tg(CD207-cre)* mice expressing BRAF V600E under control of the human Langerin promoter and *Braf*^*CA*^ control littermates were produced by crossing *Braf*^*CA*^ mice [14] with *Tg(CD207-cre)* mice [15]. *Braf*^*CA*^ mice were a gift from their developers. *Pten*^*loxP/loxP*^ mice [16], which undergo cre recombinase-mediated deletion of exon 5, were purchased from Jackson Laboratory (#006440). These mice were crossed with *Braf*^*CA*^ or with *Tg(CD207-cre)* mice to create *Braf*^*CA/CA*^*Pten*^*loxP/loxP*^ and *Pten*^*loxP/loxP*^*Tg(CD207-cre)* parental lines which, when crossed, produced *Braf*^*CA*^*Pten*^*loxP/loxP*^*Tg(CD207-cre)* mice and *Braf*^*CA*^*Pten*^*loxP/loxP*^ control littermates. Mx1-Cre mice [17] were purchased from Jackson laboratory (#003556). Male mice were used in all experiments unless otherwise indicated. All procedures involving mice were performed according to protocols approved by Dana-Farber Cancer Institute’s Institutional Animal Care and Use Committee (protocol numbers 09–083 and 11–043). Animals were housed in a specific pathogen-free ASBL-1 room in the Redstone Family Vivarium at Dana-Farber Cancer Institute. Up to five mice at a time were kept in an Animal Care Systems (Centennial, CO) Optimice sterilized microisolator cage containing BioFresh (Ferndale, WA) Performance Bedding-1/8” pelleted cellulose. Mice were maintained on a 12 hr light/dark cycle in a room controlled for temperature (72°± 2°F) and humidity (50% ± 10%). Animals had free access to water and food (Purina Prolab [St. Louis, MO] 5P76/5P75 Irradiated Diet). Enrich-n’Nest (Anderson Lab Bedding Products, Maumee, OH) bedding material was added to cages for enrichment. Animal health was assessed daily. Animals showing signs of distress such as difficulty with ambulation, persistent lethargy, or poor body condition were euthanized using carbon dioxide asphyxiation followed by cervical dislocation. Five hundred mice were used in this study.

### Genotyping

The *Braf*^*CA*^ and rearranged *Braf*^*VE*^ alleles were identified using tail snip DNA as described previously [18] using the primer pair F5’-TGA GTA TTT TTG TGG CAA CTG C and R5’-CTC TGC TGG GAA AGC GGC. A modified cycler program was used: 94° for 3 min, 43 cycles of denaturation at 94° for 30 secs, annealing at 60° for 30 sec, and elongation at 72° for 45 sec; followed by elongation for 5 min at 72°. This yields PCR products of 185 bp for wild type *Braf*, 308 bp for *Braf*^*CA*^, and 335 bp for *Braf*^*VE*^.

The *Tg(CD207-cre)* allele was identified using primers F5’-GAG GCA AAT GAT TGG CAT TCT AC and R5’-CTG GAA AAT TCA AGA AGA GCC T at 95° for 3 min, followed by 41 cycles of denaturation at 95° for 30 sec, annealing at 60° for 30 sec, elongation at 72° for 1 min; followed by elongation for 72° for 5 min. This yields a PCR product of 272 bp for the *Tg(CD207)* transgene.

*Pten* genotyping from tail DNA was performed with primers F5’-CAA GCA CTC TGC GAA CTG AG and R5’- AAG TTT TTG AAG GCA AGA TGC with PCR conditions at 94° for 3 min, 36 cycles of denaturation at 94° for 30 sec, annealing at 60° for 1 min, and elongation at 72° for 1 min; followed by elongation 72° for 2 min. PCR products of 156 bp for wild-type *Pten*, and 328 bp for *Pten*^*loxP/loxP*^ are produced. In the presence of cre recombinase, exon 5 is deleted from *Pten*^*loxP/loxP*^. To detect *Pten*^*loxP/LoxP*^ and *Pten*^*del ex5*^ in sorted cells the primers used were PTEN6637-F: 5'-TCCCAGAGTTCATACCAGGA-3', PTEN6925-R: 5'-GCAATGGCCAGTACTAGTGAAC-3', and PTEN7319-R: 5'-AATCTGTGCATGAAGGGAAC-3'. This produces a *Pten*^*loxP/loxP*^ PCR product of 650 bp and a *Pten*^*del ex5*^ PCR product of 300 bp.

### Peripheral blood analysis

Blood counts were performed with the Hemavet 950 hematology system using blood obtained by cardiac puncture from euthanized mice. Testosterone levels were analyzed at the University of Virginia Center for Research in Reproduction Ligand Assay and Analysis Core.

### Antibodies

Antibodies used in these studies included: anti-CD8a, clone 53–6.7; anti-CD11b, clone M1/70; anti- CD11c, clone N418; anti-CD103, clone 2E7; anti-I-A/I-E (MHC Class II) for flow cytometry and epidermal sections, clone M5/114.15.2 (all from Biolegend, San Diego, CA); anti-I-A/I-E for small intestine cryosections, clone 2G9 (BD Biosciences, Franklin Lakes, MJ); and anti-CD207, clone 929F3.01 (Novus Biologicals, Littleton, CA).

### Histology, immunohistochemistry, and immunofluorescence

For histological analysis of mouse tissues, samples were fixed in Bouin’s solution or 4% paraformaldehyde and paraffin embedded. Immunohistochemical staining for CD207 was performed on paraffin sections and images were obtained with the Olympus IX73 Imaging System. Immunohistochemistry for androgen receptor was performed on paraffin sections by HistoWiz (Brooklyn, NY).

To prepare epidermal sheets for immunofluorescent staining and LC quantification, ears were removed and separated into dorsal and ventral sides with tweezers. Ventral sides were floated in 3.8% ammonium thiocyanate at 37° for 30 minutes. The epidermis was separated from dermis with tweezers and fixed in ice cold acetone for 15 minutes. Epidermis was washed in PBS and blocked with PBS containing 10% rat serum for 30 minutes at room temperature. Samples were then incubated overnight at 4° with AF488-conjugated anti-I-A/I-E and AF647-conjugated anti-CD207 and mounted on slides with Vectamount anti-fade. Images were captured with a Leica SP5X laser scanning confocal microscope using the 63x objective. An average of 9.4 fields of 60 μm^2^ were captured for each mouse. The number of LCs in each field was counted using ImageJ, then checked visually and corrected for any program counting errors.

For preparation of cryosections for immunofluorescence, small intestines were flushed with cold Hank’s balanced salts solution (HBSS) using a blunt 22-gauge needle. Samples were soaked in cold 30% sucrose for 10 minutes then placed in cryomolds with Tissue-Tek OCT and frozen on a steel block cooled in liquid nitrogen. Frozen sections on glass slides were air dried for 60 minutes, fixed in ice cold acetone for 20 minutes, then blocked in PBS containing 5% rat serum for 60 minutes at room temperature. Slides were incubated with antibodies overnight at 4°C.

### Flow cytometry

Single cell suspensions of epidermis, spleen, and small intestine were prepared and analyzed on a BD LSR Fortessa or sorted on a BD FACS Aria II.

For epidermis, skin was collected from ears or from the flanks. Ears were separated into dorsal and ventral halves as described above. Flank skin was cut into small pieces and floated in 2 ml of RPMI containing 2 units/ml Dispase II neutral protease grade II (Roche Diagnostics, Risch-Rotkreutz, Switzerland, catalog #04942078001) for 90 minutes at 37°. Epidermis was separated from dermis and pulled into small pieces, then incubated with Collagenase IV (Life Technologies, Carlsbad, CA, catalog 17104019) in HBSS with calcium chloride and magnesium chloride, 10% FBS, P/S for 120 minutes at 37°. Skin was triturated by passing 3 times through a 20-gauge needle and cells were collected after passing through a 70-micron filter. Prior to staining with anti-CD207 antibodies, cells were treated with IC Fixation Buffer and Permeabilization Buffer (eBioscience/ThermoFisher, Waltham, MA).

Spleens were pressed through a 70 μm cell strainer and splenocytes were incubated for one minute in RBC Lysis Buffer (eBioscience). Splenocytes were incubated with antibodies for 30 minutes on ice in PBS containing 1% fetal bovine serum.

For small intestine, fat and mesenteric tissue were removed and small intestine was flushed with ice cold HBSS. Intestine was cut longitudinally with a scalpel and incubated in HBSS, 10% FBS, penicillin and streptomycin, 5 mM DTT for 20 minutes at 37°with shaking at 200 rpm to remove mucus. To remove epithelial cells, samples were incubated 15 minutes three times with 5 mM EDTA, HBSS, 10% fetal bovine serum, penicillin and streptomycin with shaking. Samples were washed 2x in HBSS 10 mM HEPES to remove fetal bovine serum and EDTA. Cells were then incubated with 0.2 units/ml Liberase TM (Roche Diagnostics), 40 units per ml Rnase-free Dnase I (Roche Diagnostics, catalog 04 716 728 001 in HBSS with penicillin and streptomycin for 30 minutes at 37°. DNase I was omitted when DNA was to be isolated. Liberase digestion was halted by addition of 0.5 volumes of RPMI, 10% FBS, 20 mM HEPES pH 7.4. Tissue was triturated by passing three times through an18 gauge needle and filtered through a 70 μm cell strainer.

### *Braf* mRNA analysis

Epidermal cells were sorted, and total RNA was purified using Qiagen RNeasy Micro Kit. cDNA was produced with the High Capacity cDNA Reverse Transcriptase Kit with RNase Inhibitor (Applied Biosystems, Foster City, CA). *Braf* cDNA was amplified by PCR and digested with BamHI, XbaI, or left untreated as described previously [18] to detect *Braf*, *Braf*^*CA*^, and *Braf*^*VE*^ transcription products.

### Quantitative RT-PCR

Total RNA was isolated from ventral ear skin (dermis and epidermis) by Trizol extraction. cDNA was produced by reverse transcription in the presence of RNase inhibitor. qPCR was performed on the BioRad CFX96 Real Time System using Power SYBR Green Master Mix (Applied Biosystems) with primers pairs CD207 F5’-ATGTTGAAAGGTCGTGTGGAC-3” and CD207 R5’-GGTGGTGTTCACTATCTGCATCT-3’, iCre F5’-ACAACTACCTGTTCTGCCG-3’ and iCre R5’- GCCTCAAAGATCCCTTCCAG-3’, GAPDH F5’-AGGTCGGTGTGAACGGATTTG-3’ and GAPDH R5’-TGTAGACCATGTAGTTGAGGTCA.

## Results

### Development of *Braf*^*CA*^*Tg(CD207-cre)* mice

**Fig 1:**
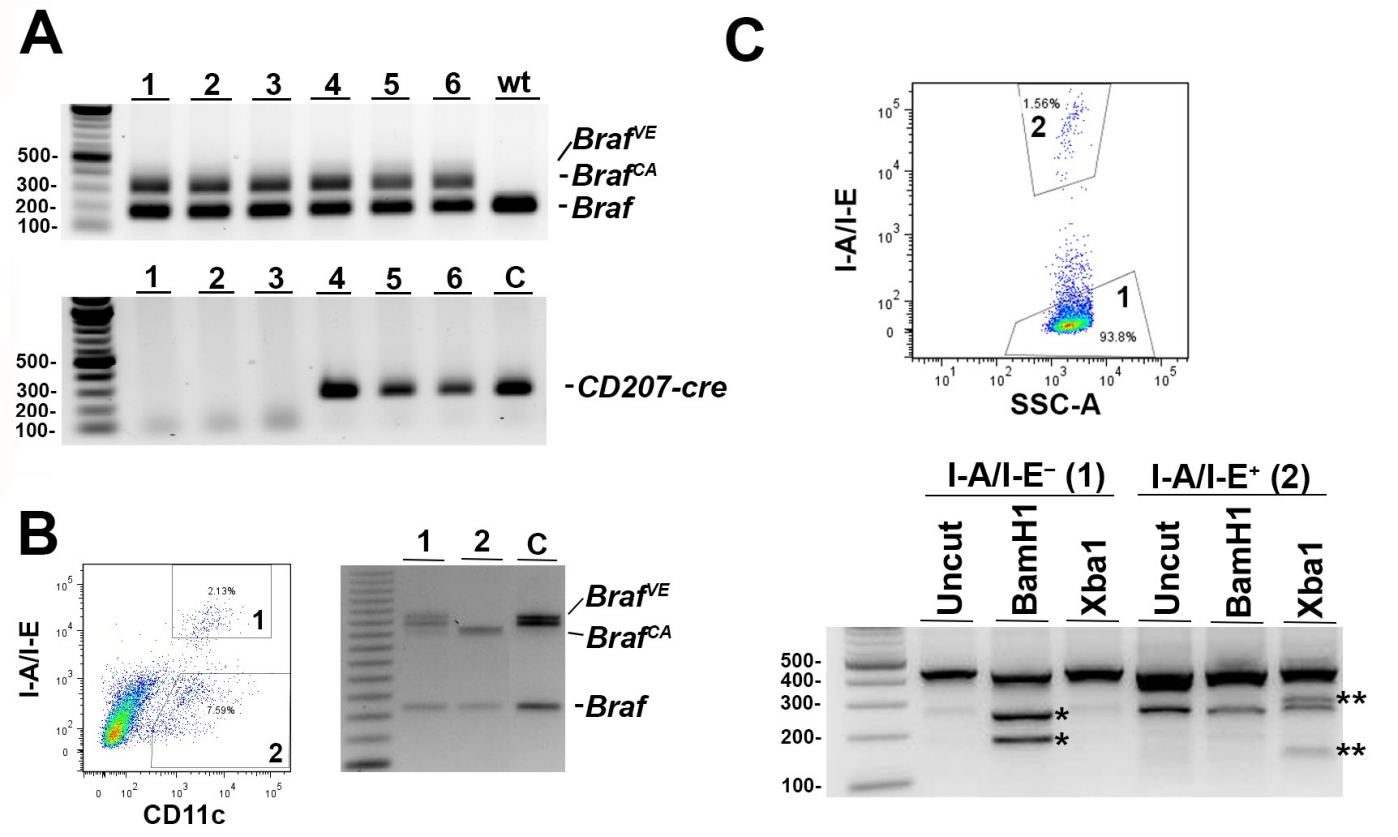
Rearrangement and expression of the rearranged *Braf*^*CA*^ allele in *Braf*^*CA*^*Tg(CD207-cre)* mice. **A. Presence of non-rearranged alleles in *Braf***^***CA***^***Tg(CD207-cre)* mice. Top panel.** PCR analysis of bulk DNA extracted from the tails of littermates (numbers 1–6) produced by a cross of *Braf*^*CA*^ with *Tg(CD207-cre)* mice, as well as a wild type mouse (wt) homozygous for wild type *Braf*. The sizes of PCR products are: 185bp for wild type *Braf*; 308bp for non-rearranged *Braf*^*CA*^; 335bp for rearranged *Braf*^*VE*^. Positions of wild type *Braf* and non-rearranged *Braf*^*CA*^ products are indicated. No product derived from *Braf*^*VE*^ was detectable. C, wild type control; **Bottom panel.** Presence of 272 bp PCR product from the *CD207-cre* allele in mice numbered 4–6. **B. Rearrangement of the *Braf***^***CA***^ **allele in Langerhans cells from *Braf***^***CA***^***Tg(CD207-cre)* mice.** Cells derived by collagenase digestion of epidermis from 34-day-old *Braf*^*CA*^*Tg(CD207-cre)* mice were sorted into CD11c^+^/I-A/I-E^-^ (region 1) and CD11c^+^/I-A/I-E^+^ (region 2) populations. PCR analysis of DNA derived from these populations for wild type *Braf* (185 bp), non-rearranged *Braf*^*CA*^ (308 bp), and rearranged *Braf*^*VE*^ (335 bp). C, positive control for the rearranged *Braf*^*VE*^ allele using bulk DNA from the spleens of *Braf*^*CA*^*Tg(Mx1-cre)* mice in which a large proportion of cells are hemizygous for the rearranged allele. **C. Expression of *Braf***^***VE***^ **mRNA in Langerhans cells from *Braf***^***CA***^***Tg(CD207-cre)* mice.** Cells derived by collagenase digestion of epidermis from 32-day-old *Braf*^*CA*^*Tg(CD207-cre)* mice were sorted into I-A/I-E^-^ (region 1) and I-A/I-E^+^ (region 2) populations. cDNA reverse transcribed from the RNA extracted from both populations was used to produce a 425 bp PCR product spanning the wild type and transgenic *Braf* loci (I-A/I-E^-^, uncut). Non-rearranged *Braf*^*CA*^ contains a BamH1 site and digestion of the PCR product from the non-rearranged allele produces fragments of 247 bp and 178 bp (I-A/I-E^-^, BamH1, *) which are not present in the BamH1 digestion of the I-A/I-E^+^ cells. Rearranged *Braf*^*VE*^ loses the BamH1 site but gains an XbaI site and digestion of the PCR product from the rearranged allele produces fragments of 286 bp and 139 bp (I-A/I-E^+^, XbaI, **) which are not present in the XbaI digestion of the I-A/I-E^-^ cells. (The PCR product of approximately 260 bp in all three lanes from I-A/I-E^+^ cells is a contaminant which is also present in lower amounts in the three lanes from I-A/I-E^-^ mice).

To test the effect of BRAF V600E expression in Langerin-expressing cells *in vivo* we crossed mice carrying the conditional *BRAF* allele, *Braf*^*CA*^, with mice carrying a bacterial artificial chromosome in which the human *Langerin* locus was modified by inserting a cDNA encoding cre recombinase at the translation initiation site (*Tg*(*CD207-cre*)). The non-rearranged *Braf*^*CA*^ allele expresses wild-type BRAF under control of the endogenous mouse promoter. In cells in which the human Langerin promoter is active, expression of cre recombinase results in rearrangement of the allele to form *Braf*^*VE*^, which encodes BRAF V600E. Several mice carrying both the *Braf*^*CA*^ and *Tg(CD207-cre)* alleles were identified using DNA extracted from tail snips but no *Braf*^*VE*^ could be detected (Fig 1A). This is most likely a consequence of the small number of Langerin-expressing cells in tail tissues. Therefore, to test for cre-mediated *Braf*^*CA*^ rearrangement, we analyzed DNA from epidermal Langerhans cells (LCs) isolated as described in Materials and Methods. I-A/I-E^+^/CD11c^+^ LCs from mice carrying both the *Braf*^*CA*^ and *Tg(CD207-cre)* alleles showed rearrangement of *Braf*^*CA*^ to produce *Braf*^*VE*^ (Fig 1B). Mice carrying only *Braf*^*CA*^ showed no such rearrangement, nor did cells that were I-A/I-E^-^/CD11c^+^ from mice carrying both alleles. In addition, I-A/I-E^+^ cells expressed an mRNA specific for the rearranged *Braf*^*VE*^ allele (Fig 1C) which was not expressed in I-A/I-E^-^ cells from the same mice. Thus, *Braf*^*CA*^ is rearranged in the LCs of *Braf*^*CA*^*Tg(CD207-cre)* mice and expresses an mRNA encoding BRAF V600E. Unfortunately, the VE1 antibody directed against human BRAF V600E provided no specific immunohistochemical staining in these or control mouse tissues, and so we were unable to document BRAF V600E protein expression. Careful literature review revealed no examples of specific staining using VE1 to detect BRAF V600E in mouse tissues suggesting that this is a commonly encountered problem.

### Macroscopic phenotype of *Braf*^*CA*^*Tg(CD207-cre)* mice

**Fig 2:**
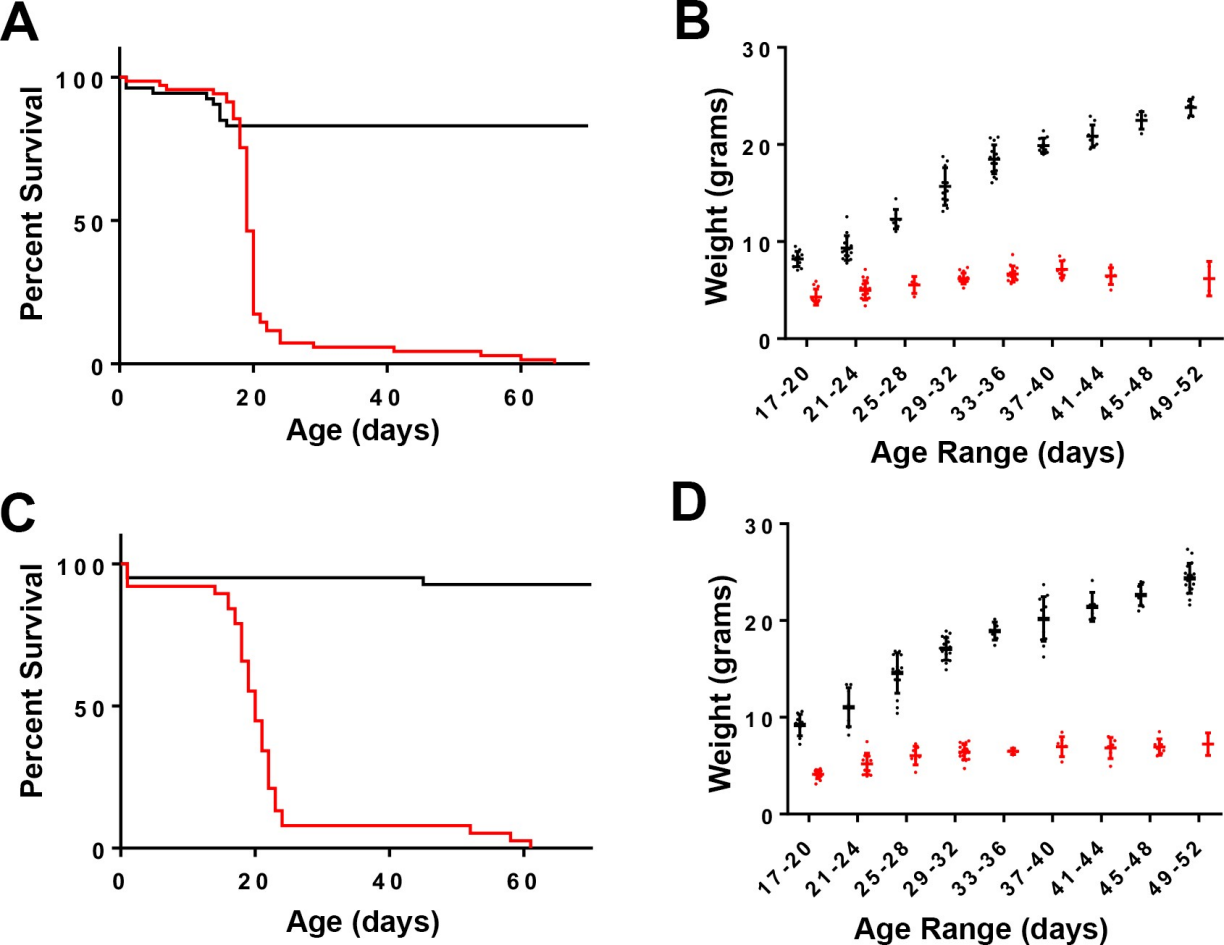
Survival and weights of genetically modified mice. **A.** Kaplan-Meier plot showing proportional survival of *Braf*^*CA*^ (black line, n = 53) and *Braf*^*CA*^*Tg(CD207-cre)* (red line, n = 69) littermates. The median survival of *Braf*^*CA*^*Tg(CD207-cre)* mice was 19 days. P < 0.0001 by log-rank test. **B.** Weights of *Braf*^*CA*^ (black dots) and *Braf*^*CA*^*Tg(CD207-cre)* (red dots) mice. Horizontal bars show means. p ≤ 8.9 x 10^−7^ by t-test for all time points. **C.** Kaplan-Meier plot showing proportional survival of *Braf*^*CA*^*Pten*^*loxP/loxP*^ mice (black line, n = 41) and *Braf*^*CA*^*Pten*^*loxP/loxP*^*Tg(CD207-cre)* littermates (red line, n = 38). The median survival of *Braf*^*CA*^*Pten*^*loxP/loxP*^*Tg(CD207-cre)* mice was 20 days. P < 0.0001 by log-rank test. **D.** Weights of *Braf*^*CA*^*Pten*^*loxP/loxP*^ (black dots) and *Braf*^*CA*^*Pten*^*loxP/loxP*^*Tg(CD207-cre)* (red dots) mice. Horizontal bars show means. p ≤ 2.8 x 10^−7^ by t-test for all time points.

We consistently observed a smaller than expected number *Braf*^*CA*^*Tg(CD207-cre)* progeny from crosses involving mice heterozygous or homozygous for the *Braf*^*CA*^ with *Tg*(*CD207-cre*) alleles. For example, among 307 evaluable progeny from several such crosses, 124 *Braf*^*CA*^*Tg(CD207-cre)* mice were expected but only 24 were observed when we performed genotyping at 3–4 weeks of age (p < 0.0001 by two-sided exact binomial test). However, by carefully monitoring all litters, we determined that *Braf*^*CA*^*Tg(CD207-cre)* mice were born alive in expected numbers but had a median survival of only 19 days and all mice of that genotype were dead by 65 days of age (Fig 2A).

**Fig 3:**
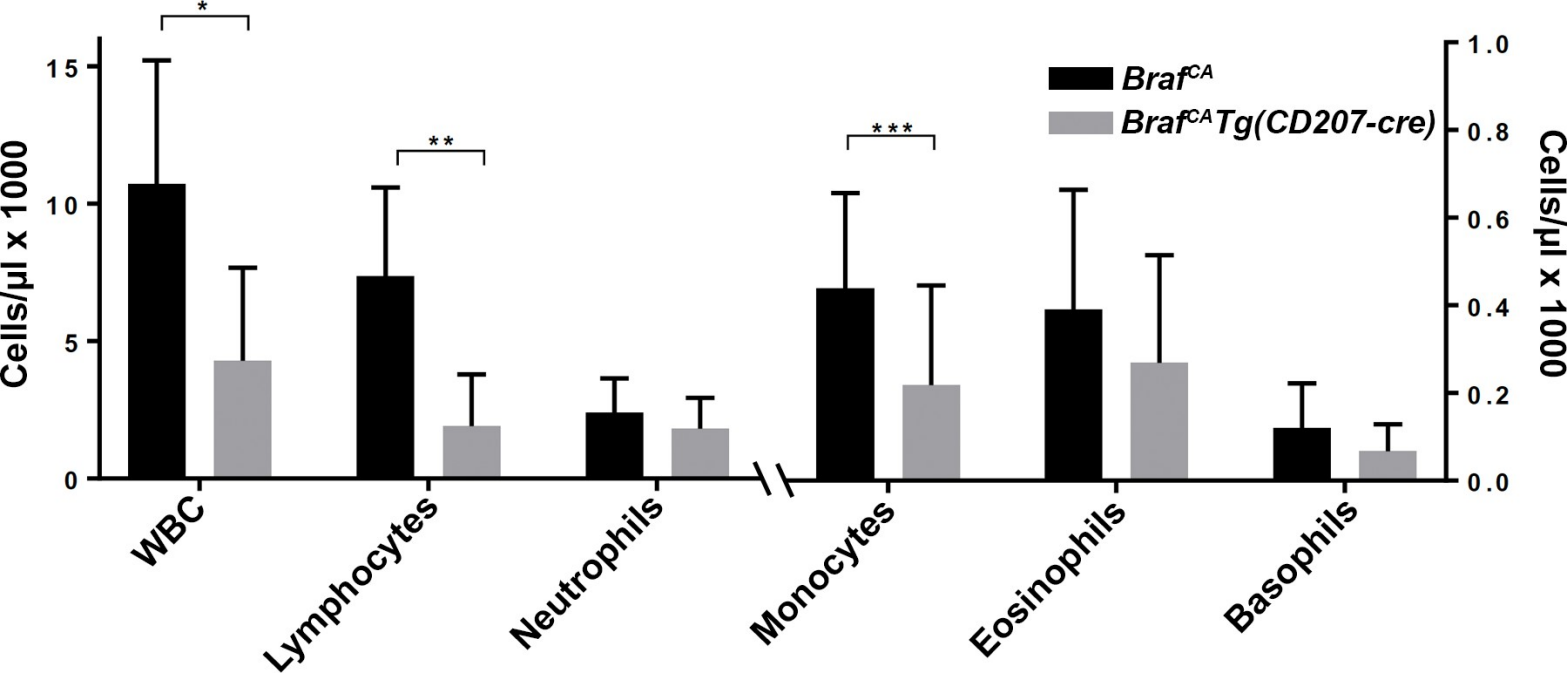
Peripheral blood counts in *Braf*^*CA*^*Tg(CD207-cre)* mice. Total white blood count (WBC) and differential count in *Braf*^*CA*^ and *Braf*^*CA*^*Tg(CD207-cre)* mice aged 20 to 39 days. WBC, lymphocyte, and neutrophil counts are shown on the left ordinate; monocyte, eosinophil, and basophil counts are shown on the right ordinate. Results are shown as the mean and standard deviation of 19 *Braf*^*CA*^ and 13 *Braf*^*CA*^*Tg(CD207-cre)* mice. Comparisons made by two-sided Welch t-test: *, p = 7.27 x 10^−5^; **, p = 1.45 x 10^−6^; ***, p = 0.0113.

**Fig 4:**
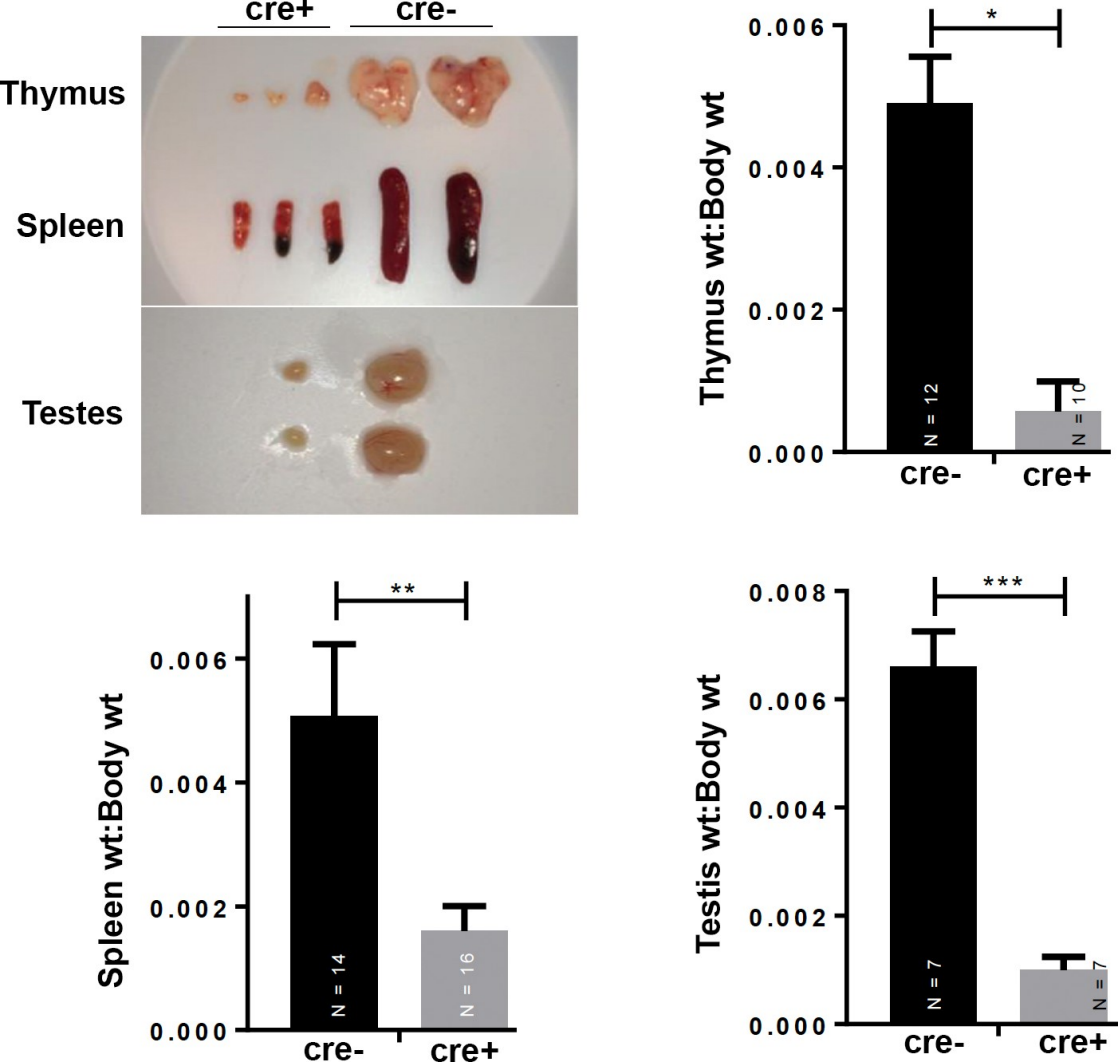
Organ size in *Braf*^*CA*^ and *Braf*^*CA*^*Tg(CD207-cre)* mice. Photographs of representative thymi and spleens, from 31 day old mice, and testes from 39 day old *Braf*^*CA*^*Tg(CD207-cre)* (cre+) and *Braf*^*CA*^ (cre-) mice. Bar graphs indicate the mean ratio of organ weight (thymus, spleen, testis) to body weight for the indicated *Braf*^*CA*^ (cre-) and *Braf*^*CA*^*Tg(CD207-cre)* (cre+) mice. Thymi and spleens were from 30 to 50 day old mice. Testes were from mice aged 30 to 41 days. Comparisons made by two-sided Welch t-test: *, p = 8.85 x 10^−14^; **, p = 1.43 x 10^−8^; ***, p = 3.69 x 10^−8^.

Although 50% of *Braf*^*CA*^*Tg(CD207-cre)* mice died by 19 days of age a significant proportion could be weaned and survived for nearly two months. At birth, *Braf*^*CA*^*Tg(CD207-cre)* mice appeared similar to their *Braf*^*CA*^ littermates but gained little to no weight after weaning (Fig 2B). Thorough anatomic and histological examination revealed no obvious cause for their low weight and early death. These mice had a 60% lower total white blood count than controls predominantly due to a 73% reduction in lymphocytes and a lesser reduction in monocytes (Fig 3). *Braf*^*CA*^*Tg(CD207-cre)* mice also had smaller thymi, spleens, and testes compared to controls as determined by visual inspection and weight (Fig 4).

### Microscopic phenotype of *Braf*^*CA*^*Tg(CD207-cre)* mice

**Fig 5:**
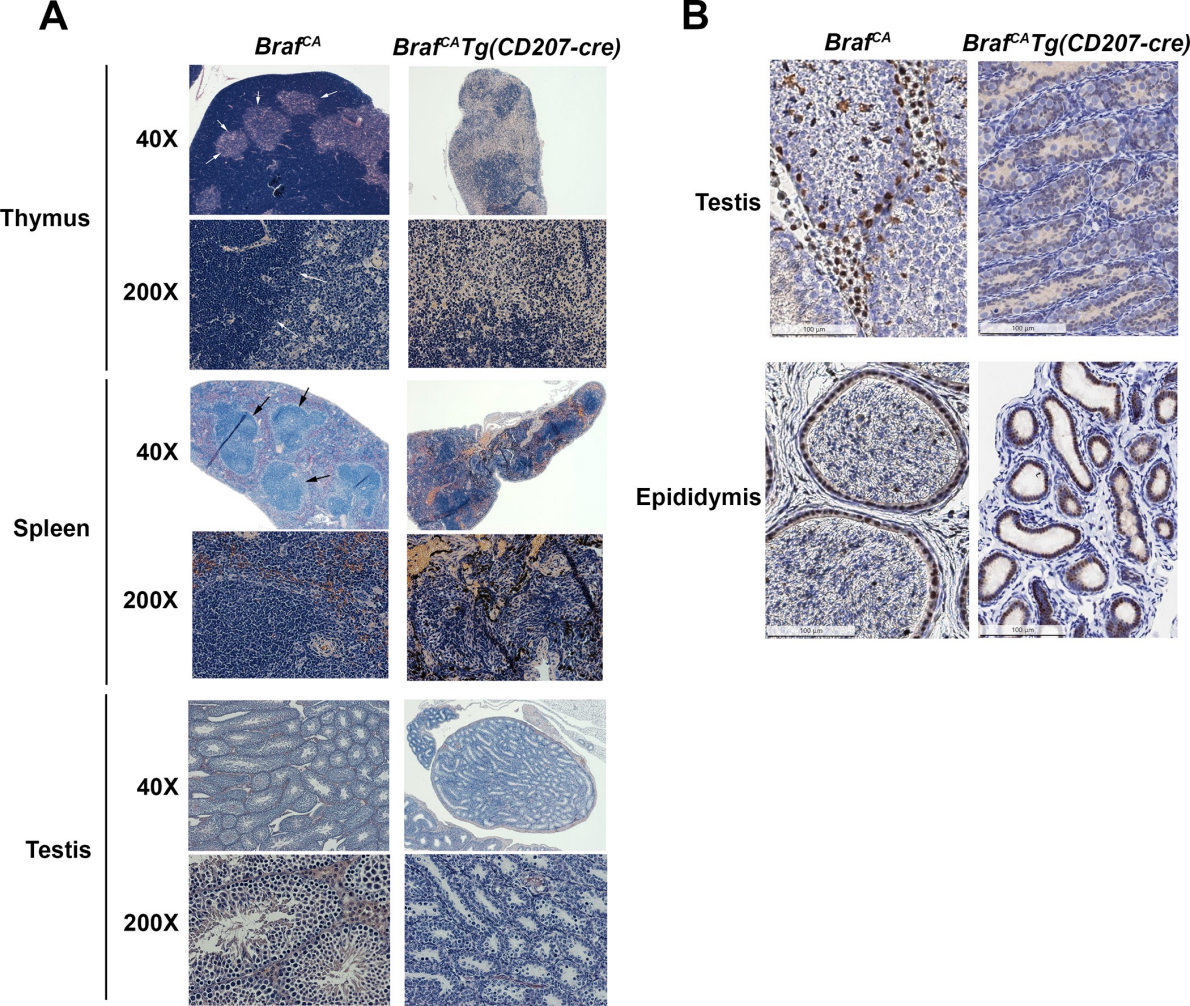
Histology of thymus, spleen, and testis in *Braf*^*CA*^ and *Braf*^*CA*^*Tg(CD207-cre)* mice. **A**. **Histology of thymus, spleen, and testis**. Hematoxylin and eosin staining of representative microscopic sections of thymus, spleen, and testis from *Braf*^*CA*^ and *Braf*^*CA*^*Tg(CD207-cre)* mice at 40X and 200X magnification. The sharp cortico-medullary border in the thymus of *Braf*^*CA*^ mice (arrows) is obscured in the thymus of *Braf*^*CA*^*Tg(CD207-cre)* 19 day old female mice. Similarly, the sharp border between red pulp and white pulp in the spleen of *Braf*^*CA*^ mice (arrows) is obscured in the spleen of *Braf*^*CA*^*Tg(CD207-cre)* 36 day old male mice. Spermatozoa which are visible in the testis of 36 day old *Braf*^*CA*^ mice are absent from the testis of 42 day old *Braf*^*CA*^*Tg(CD207-cre)* mice. Original magnifications, 40X and 200X as indicated. **B. Androgen receptor expression in testis and epididymis.** Immunohistochemical staining for androgen receptor of testis and epididymis from *Braf*^*CA*^ and *Braf*^*CA*^*Tg(CD207-cre)* mice. Androgen receptor staining is apparent in Leydig and some Sertoli cells in *Braf*^*CA*^ mice but not *Braf*^*CA*^*Tg(CD207-cre)* mice (both 58 days of age). However, androgen receptor staining is present in the epididymis of both genotypes at the same age. Scale bars are shown.

Histological analysis of the thymus showed absence of a well-defined cortical-medullary border in *Braf*^*CA*^*Tg(CD207-cre)* mice with a decrease in numbers of cortical lymphocytes (Fig 5A). Spleens from *Braf*^*CA*^*Tg(CD207-cre)* mice showed a loss of distinct zones of red and white pulp (Fig 5A). Leydig cells and mature spermatozoa were absent from testes of *Braf*^*CA*^*Tg(CD207-cre)* mice (Fig 5A). No accumulation of histiocytic cells was observed in any organ from *Braf*^*CA*^*Tg(CD207-cre)* mice at any age.

Although Leydig cells appeared to be absent from the testes of *Braf*^*CA*^*Tg(CD207-cre)* mice, serum testosterone levels were only 46% lower than *Braf*^*CA*^*Tg* mice (41.3 ± 7.9 ng/dL vs. 76.0 ± 25.1 ng/dL, p = 0.04) suggesting either an alternative source of hormone or only a partial loss of Leydig cells. However, immunohistochemical staining for androgen receptor showed no specific staining in the testes of *Braf*^*CA*^*Tg(CD207-cre)* mice indicating a near complete absence of Leydig cells (Fig 5B). Epididymal cells did show staining in these mice indicating that they did not have a global loss of androgen receptor (Fig 5B).

**Fig 6:**
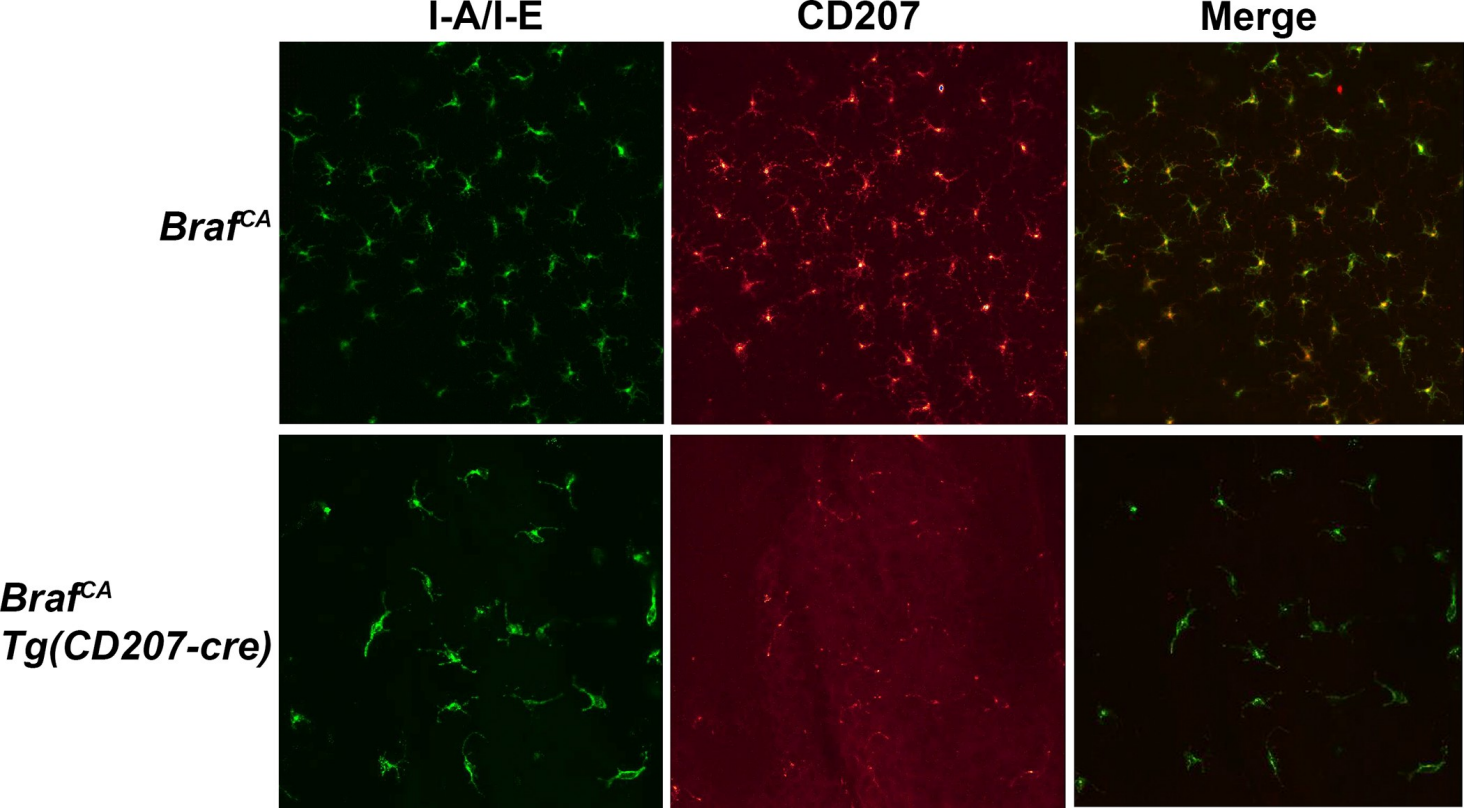
Epidermal Langerhans cells identified by immunofluorescence in *Braf*^*CA*^ and *Braf*^*CA*^*Tg(CD207-cre)* mice. Epidermis was prepared from the ears of 43 day old mice of the indicated genotype as described in Materials and Methods. Immunofluorescence imaging was performed using antibodies directed against MHC Class II (I-A/I-E, green) and CD207 (red). Merged images are also shown indicating that nearly all I-A/I-E^+^ cells are also CD207^+^ in *Braf*^*CA*^ mice, identifying these cells as Langerhans cells. In *Braf*^*CA*^*Tg(CD207-cre)* mice, most Langerhans cells have reduced CD207 expression.

**Fig 7:**
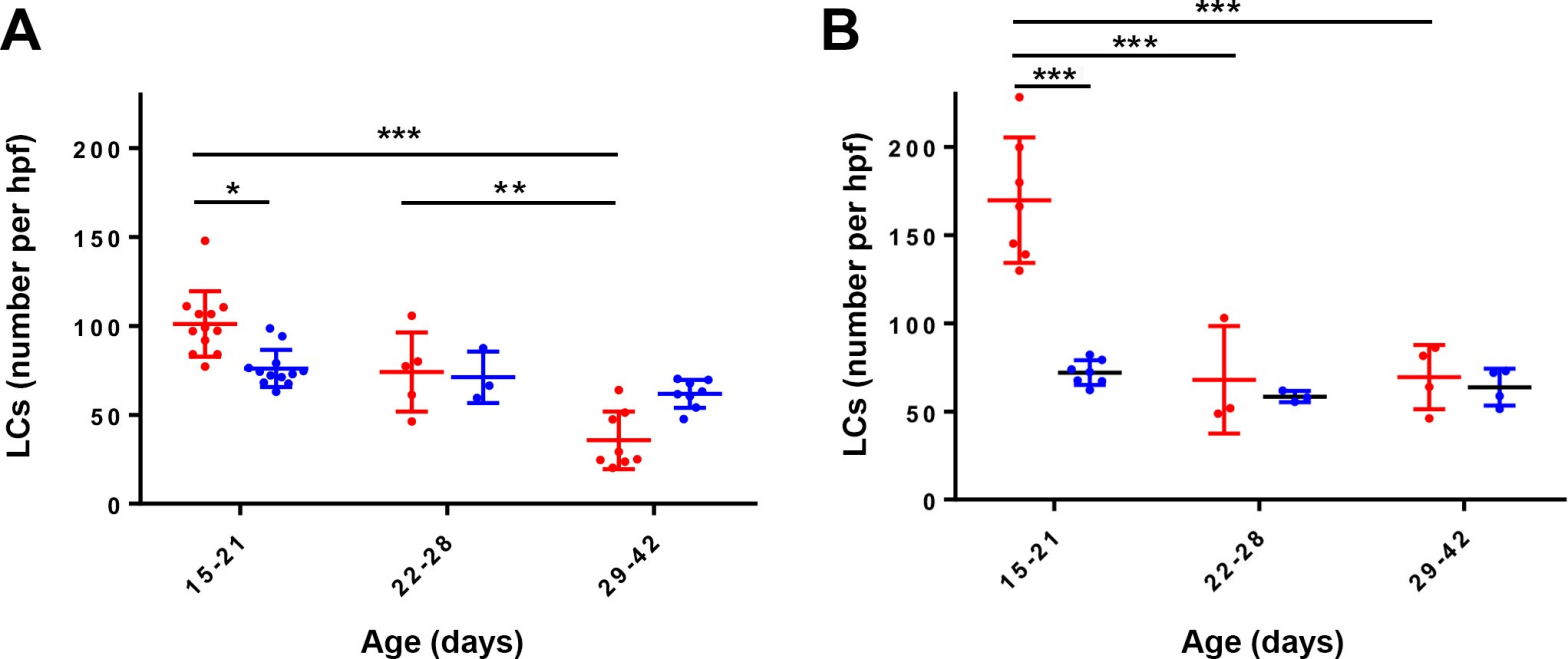
Quantitation of epidermal Langerhans cells in genetically modified mice. **A. Epidermal Langerhans cells in *Braf***^***CA***^ **and *Braf***^***CA***^***Tg(CD207-cre)* mice.** Epidermal sheets were prepared from *Braf*^*CA*^ (blue dots) and *Braf*^*CA*^*Tg(CD207-cre)* (red dots) mice of the indicated ages and stained with fluorescence-conjugated antibodies against I-A/I-E. LCs were counted as described in Materials and Methods. Mean is plotted as the horizontal bar for each group and error bars indicate standard deviation. All comparisons were determined using a mixed model with a random effect that included mutational status, age, and an interaction term to analyze repeated measures over time. A Tukey-Kramer adjustment for p-values was then used to test the difference between mutational status and time. *, p = 0.0309; **, p = 0.0062; ***, p < 0.001. **B. Epidermal Langerhans cells in *Braf***^***CA***^***Pten***^***loxP/loxP***^ **and *Braf***^***CA***^***Pten***^***loxP/loxP***^***Tg(CD207-cre)* mice.** Epidermal sheets were prepared from *Braf*^*CA*^*Pten*^*loxP/loxP*^ (blue dots) and *Braf*^*CA*^*Pten*^*loxP/loxP*^*Tg(CD207-cre)* (red dots) mice of the indicated ages and stained with fluorescence-conjugated antibodies against I-A/I-E. LCs were counted as described in Materials and Methods. Comparisons were made using a model similar to that described in **A**. ***, p< 0.001.

To examine the effect of BRAF V600E expression on LCs, we identified epidermal I-A/I-E^+^/CD207^+^ cells by immunofluorescence (Fig 6) in *Braf*^*CA*^ and *Braf*^*CA*^*Tg(CD207-cre)* mice and measured their abundance. *Braf*^*CA*^*Tg(CD207-cre)* mice had significantly higher numbers of LCs than controls at 15–21 days of age but that number steadily decreased so that the number of LCs in *Braf*^*CA*^*Tg(CD207-cre)* was the same as in control *Braf*^*CA*^ mice at 22–28 days of age, and lower than in those controls by 29–42 days of age (Fig 7A). Thus, expression of BRAF V600E in cells in which the human Langerin promoter is active leads to an early expansion of cutaneous LCs within 1–3 weeks of birth but their number steadily declines thereafter. The absence of sustained LC proliferation in *Braf*^*CA*^*Tg(CD207-cre)* is not due to loss of rearranged *Braf*^*VE*^ since the rearranged allele was detected in epidermal LCs from these mice (Fig 1B).

**Fig 8:**
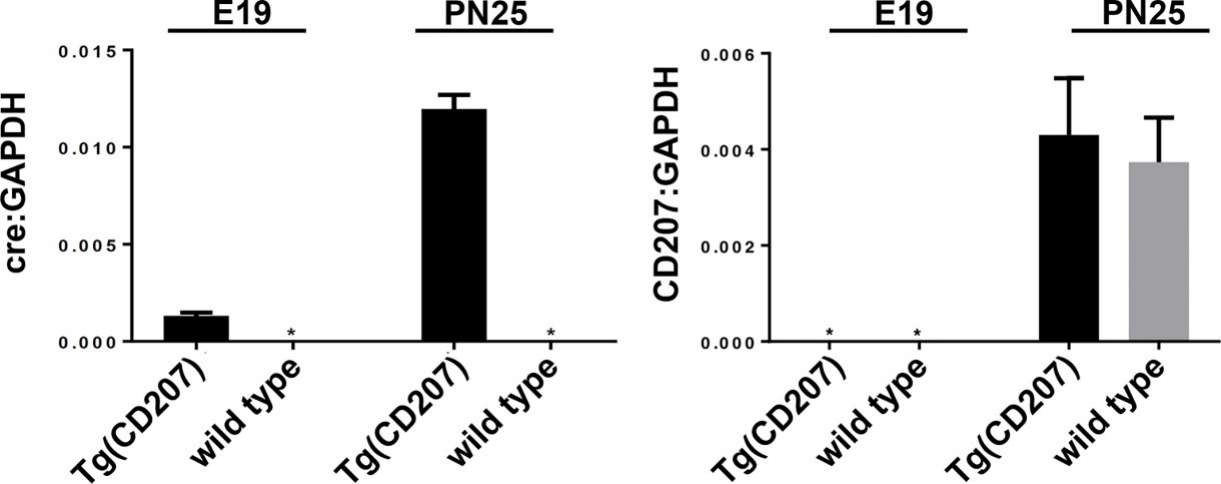
Expression of human CD207-driven cre recombinase occurs earlier in development than expression of endogenous murine CD207. RNA was extracted from trunk skin of embryos of the indicated genotype at post-coital day 19 (E19) and from the ear skin of mice of the indicated genotype at 25 days of age postnatal (PN25). RNA was analyzed by quantitative RT-PCR for the expression of cre recombinase, CD207, and GAPDH. The ratios of C_t_ for cre recombinase to C_t_ for GAPDH are shown in the plot on the left; the ratios of C_t_ for CD207 to C_t_ for GAPDH are shown in the plot on the right. Results were combined from three mice for each age and genotype. The E19 *Tg(CD207)* group had 2 males and one female, while the wild type group had 2 females and one male. *, no expression detected.

This phenotype is strikingly different from the mild LCH-like phenotype reported by Berres et al. in which cre-recombinase expression was driven by the murine Langerin promoter [13]. To test whether the difference in phenotypes between *Braf*^*CA*^*Tg(CD207-cre)* and the Berres et al. mice might be due to differences in the temporal pattern of human *versus* murine *Langerin* expression, we used quantitative RT-PCR to compare the levels of cre recombinase and endogenous CD207 mRNA expression in *Tg(CD207-cre)* mice at two different time points. Cre recombinase mRNA expression driven by the human Langerin promoter was detectable in day 19 post-coital embryos (designated E19 in Fig 8) while expression of CD207 driven by the endogenous Langerin promoter was not. Both promoters were active at postnatal day 25 (designated PN25 in Fig 8). This temporal difference in human *versus* murine Langerin expression combined with differences in tissue-specific expression, discussed below, may account for the phenotypic differences in these two models.

### Development of *Braf*^*CA*^*Pten*^*loxP/loxP*^*Tg(CD207-cre)* mice

**Fig 9:**
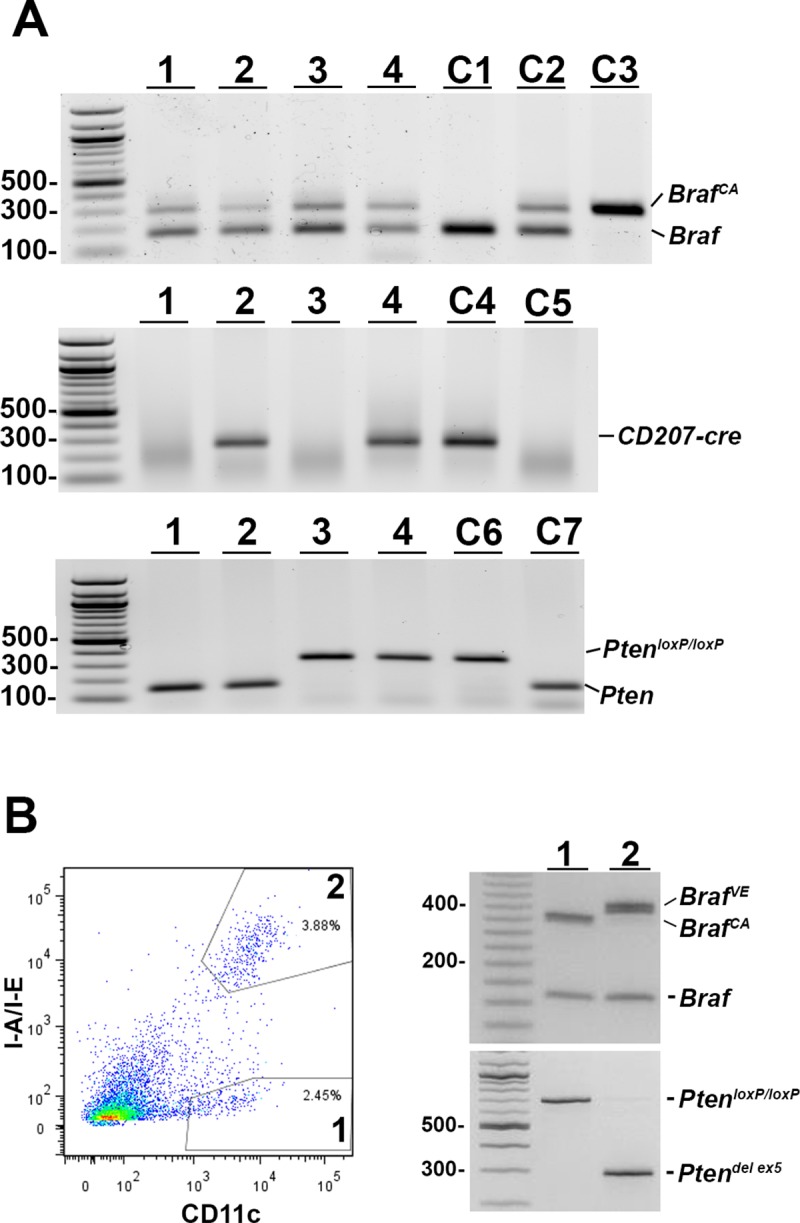
Presence and rearrangement of *Braf* and *Pten* alleles in *Braf*^*CA*^*Pten*^*loxP/loxP*^*Tg(CD207-cre)* mice. **A. Presence of non-rearranged alleles in tail-cut DNA of *Braf***^***CA***^***Pten***^***loxP/loxP***^***Tg(CD207-cre)* mice. Top panel,** DNA was extracted from tails of mice numbered 1–4 and analyzed by PCR for the presence of wild type *Braf* (product size 185 bp), non-rearranged *Braf*^*CA*^ (308 bp), and *Braf*^*VE*^ (335 bp). Only the wild type and non-rearranged alleles are detectable in bulk DNA. C1, C2, and C3 are control DNAs from mice homozygous for wild type *Braf*, mice heterozygous for wild type *Braf* and *Braf*^*CA*^, and mice homozygous for *Braf*^*CA*^, respectively. **Middle panel,** DNA from the tails of mice 1–4 was analyzed for the presence of the *CD207-cre* allele. The expected product of 272 bp was observed in mice 2 and 4. C4 and C5 are control DNAs from mice known to carry the *CD207-cre* allele, and wild type mice, respectively. **Bottom panel,** DNA from the tails of mice 1–4 was analyzed for the presence of the *Pten*^*loxP/loxP*^ allele. The expected product of 328 bp was observed in mice 3 and 4, and the PCR product of 156 bp derived from the wild type *Pten* allele was observed in mice 1 and 2. C6 and C7 are control DNAs from mice known to carry the *Pten*^*loxP/loxP*^ allele, and wild type mice, respectively. **B. Rearranged *Braf* and *Pten* alleles in epidermal LCs of *Braf***^***CA***^***Pten***^***loxP/loxP***^***Tg(CD207-cre)* mice.** Cells were isolated from the epidermis of a 47-day-old *Braf*^*CA*^*Pten*^*loxP/loxP*^*Tg(CD207-cre)* mouse as described in Materials and Methods and sorted into I-A/I-E^-^/CD11c^+^ (1) and I-A/I-E^+^/CD11c^+^ (2) populations (left panel). DNA was extracted from each cell population and analyzed by PCR for the presence of *BRAF* and *Pten* alleles. The LCs (population 1) showed rearranged *BRAF*^*VE*^ and *Pten*^*del ex5*^ alleles which were absent from the non-LC cells (population 2).

The progressive loss of epidermal LCs in *Braf*^*CA*^*Tg(CD207-cre)* mice raises the possibility that the expression of BRAF V600E in those cells causes oncogene-induced senescence or apoptosis. By analogy to other MAPK-driven neoplasms, oncogene-induced proliferation may require loss of tumor suppressor genes [10, 11]. To test this idea, we crossed *Braf*^*CA*^*Tg(CD207-cre)* mice with mice carrying a *Pten* allele in which exon 5 is floxed, and performed intercrosses to develop *Braf*^*CA*^*Pten*^*loxP/loxP*^*Tg(CD207-cre)* mice which are homozygous for floxed *Pten* and also carry the *Braf*^*CA*^ allele and *Tg(CD207-cre)*. Similar to *Braf*^*CA*^*Tg(CD207-cre)* mice, the proportion of CD207-expressing cells in the tail tissues used for DNA analysis was too low to detect rearranged alleles (Fig 9A). However, rearranged *Braf*^*VE*^ and *Pten*^*del ex5*^ alleles were detected in sorted epidermal Langerhans cells (Fig 9B).

### Comparison of *Braf*^*CA*^*Pten*^*loxP/loxP*^*Tg(CD207-cre)* to *Braf*^*CA*^*Tg(CD207-cre)* mice

**Fig 10:**
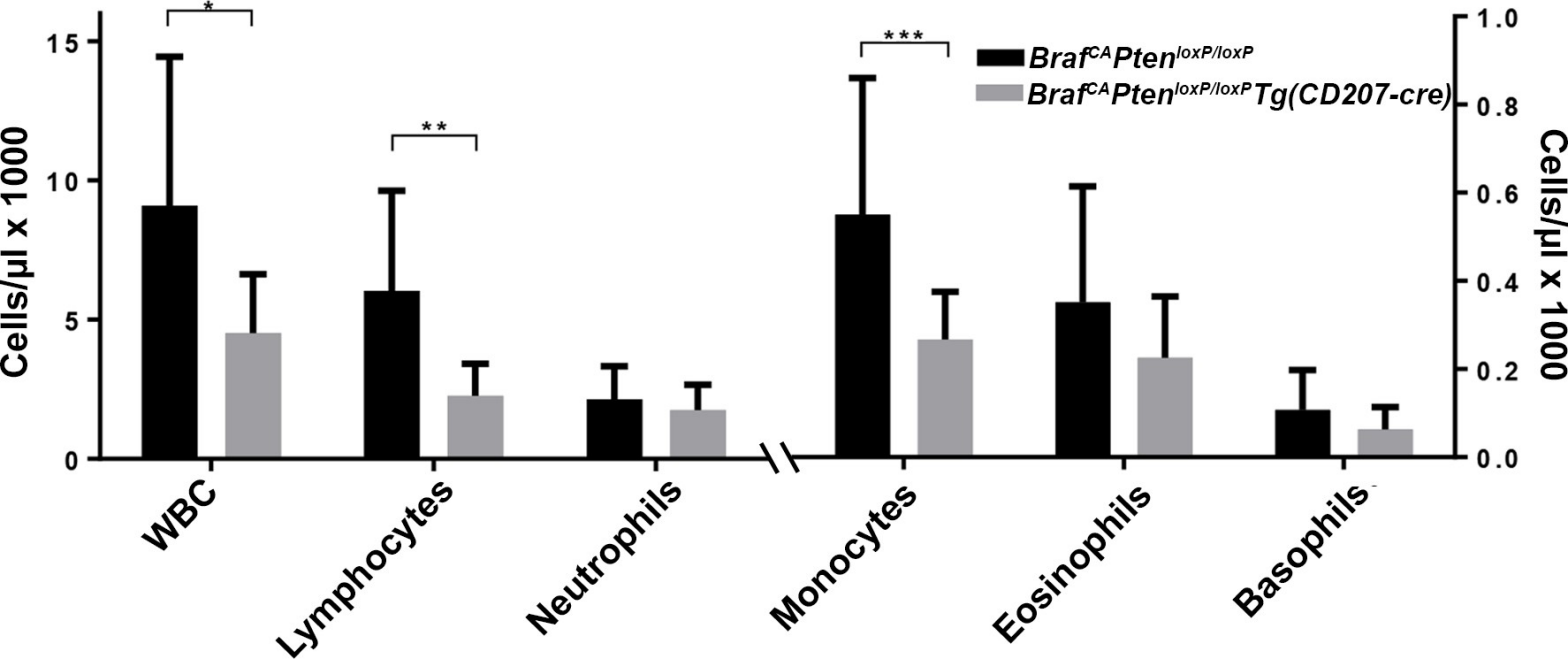
Peripheral blood counts in *Braf*^*CA*^*Pten*^*loxP/loxP*^*Tg(CD207-cre)* mice. Total white blood count (WBC) and differential count in *Braf*^*CA*^ and *Braf*^*CA*^*Pten*^*loxP/loxP*^*Tg(CD207-cre)* mice. WBC, lymphocyte, and neutrophil counts are shown on the left ordinate; monocyte, eosinophil, and basophil counts are shown on the right ordinate. Results are shown as the mean and standard deviation of 8 *Braf*^*CA*^ and 8 *Braf*^*CA*^*Pten*^*loxP/loxP*^*Tg(CD207-cre)* mice, aged 19 to 31 days. Comparisons made by two-sided Welch t-test: *, p = 0.0512; **, p = 0.0213; ***, p = 0.0487.

**Fig 11:**
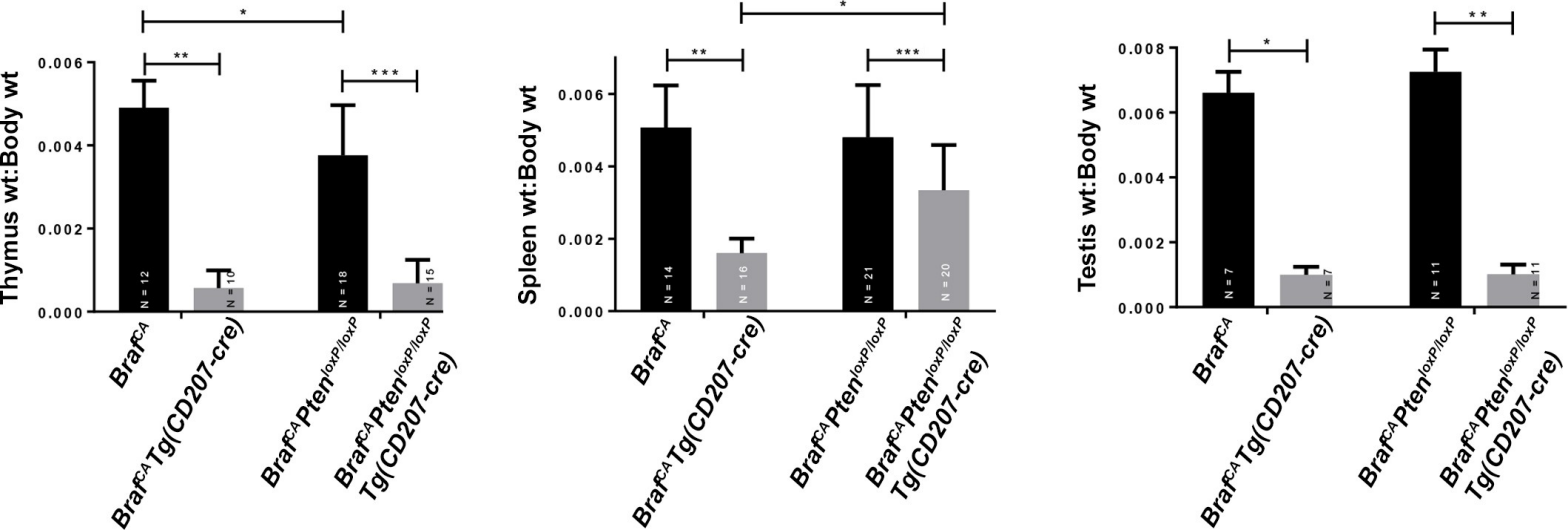
Organ size in genetically modified mice. Thymus, spleen, and testis weights were determined relative to the body weights of the mice of the indicated genotypes from which they were derived. The decreased weights of thymus and testis in *Braf*^*CA*^*Pten*^*loxP/loxP*^*Tg(CD207-cre)* compared to controls was similar to the decrease seen in *Braf*^*CA*^*Tg(CD207-cre)* mice. However, spleen weight was closer to controls in *Braf*^*CA*^*Pten*^*loxP/loxP*^*Tg(CD207-cre)* mice compared to *Braf*^*CA*^*Tg(CD207-cre)* mice (middle panel). Numbers of mice tested are indicated. *Braf*^*CA*^*Pten*^*loxP/loxP*^*Tg(CD207-cre)* and *Braf*^*CA*^*Tg(CD207-cre)* mice ranged in age from 28 to 54 days. *Braf*^*CA*^*Tg(CD207-cre)* and *Braf*^*CA*^ *mice are the same as shown in Fig 4.* Comparisons made by two-sided Welch t-test: left panel (thymus): *, p = 2.26 x 10^−3^; **, p = 8.85 x 10^−14^; ***, p = 7.02 x 10^−10^; middle panel (spleen): *, p = 4.96 x 10^−6^; **, p = 1.43 x 10^−8^; ***, p = 1.30 x 10^−3^; right panel (testis): *, p = 3.69 x 10^−8^; **, p = 1.98 x 10^−13^.

Like *Braf*^*CA*^*Tg(CD207-cre)* mice, *Braf*^*CA*^*Pten*^*loxP/loxP*^*Tg(CD207-cre)* mice have a median survival of 20 days and very few survive to 60 days of age (Fig 2C). Also similar to *Braf*^*CA*^*Tg(CD207-cre)* mice, surviving *Braf*^*CA*^*Pten*^*loxP/loxP*^*Tg(CD207-cre)* mice have lower weights than controls (Fig 2D) and lower numbers of circulating lymphocytes and monocytes but a non-significant decrease in total white blood count (Fig 10). Finally, although the thymi and testes of *Braf*^*CA*^*Pten*^*loxP/loxP*^*Tg(CD207-cre)* mice are reduced in size to the same extent as those of *Braf*^*CA*^*Tg(CD207-cre)* mice, spleen size is not reduced as much (Fig 11). (We have reported as controls the phenotypes of *Braf*^*CA*^*Pten*^*loxP/loxP*^ mice rather than *Pten*^*loxP/loxP*^*Tg(CD207-cre)* mice because *Pten*^*loxP/loxP*^*Tg(CD207-cre)* mice were indistinguishable from *Pten*^*loxP/loxP*^ mice in these experiments.)

**Fig 12:**
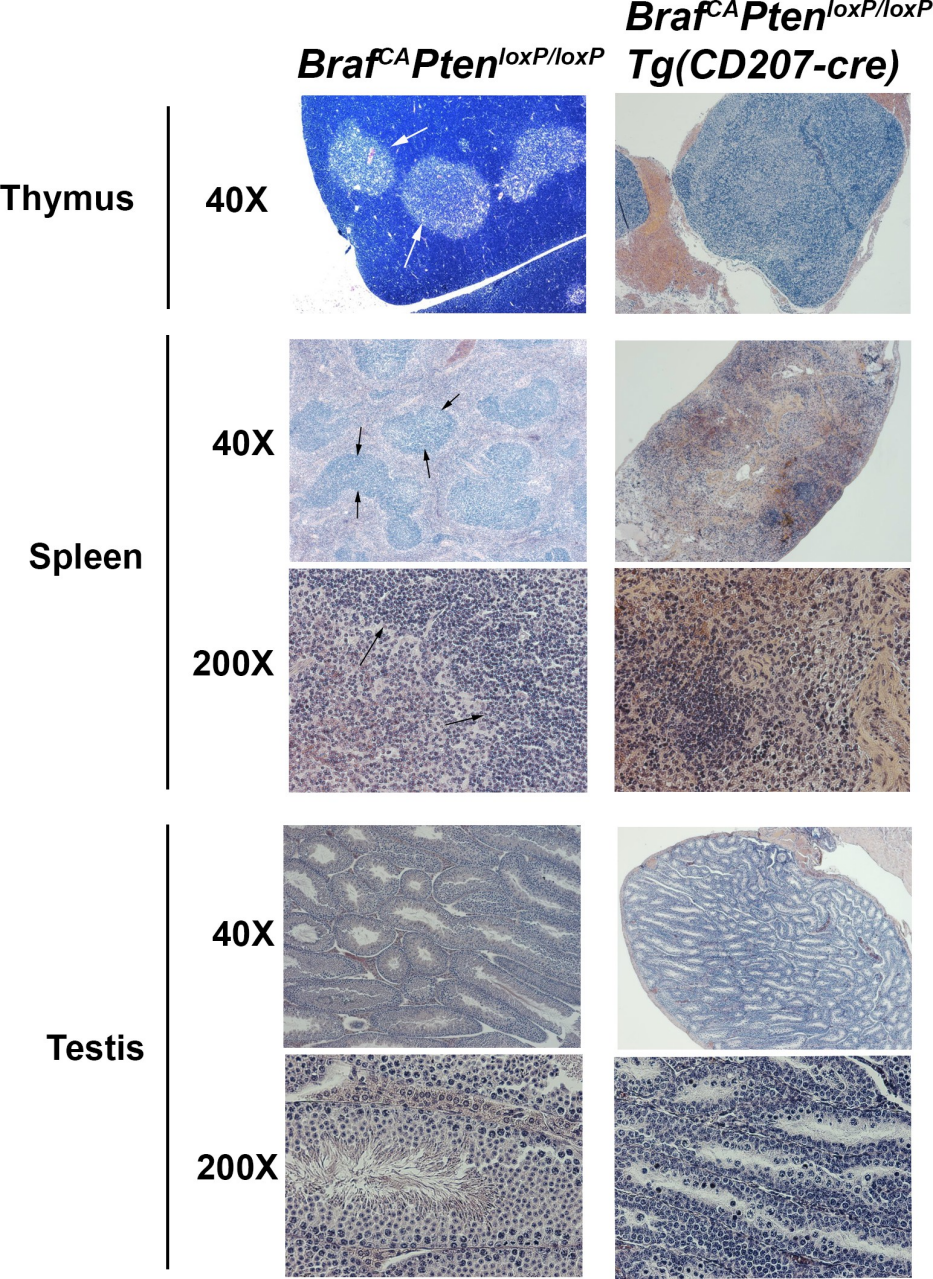
Histology of thymus, spleen, and testis in *Braf*^*CA*^*Pten*^*loxP/loxP*^ and *Braf*^*CA*^*Pten*^*loxP/loxP*^*Tg(CD207-cre)* mice. Hematoxylin and eosin staining of representative microscopic sections from *Braf*^*CA*^*Pten*^*loxP/loxP*^ and *Braf*^*CA*^*Pten*^*loxP/loxP*^*Tg(CD207-cre)* mice at 40X and 200X magnification. Thymus, spleen, and testis were from mice aged 21, 61, and 58 days respectively. The sharp cortico-medullary border in the thymus of *Braf*^*CA*^*Pten*^*loxP/loxP*^ mice (arrows) is obscured in the thymus of *Braf*^*CA*^*Pten*^*loxP/loxP*^*Tg(CD207-cre)* mice. Similarly, the sharp border between red pulp and white pulp in the spleen of *Braf*^*CA*^*Pten*^*loxP/loxP*^ mice (arrows) is obscured in the spleen of *Braf*^*CA*^*Pten*^*loxP/loxP*^*Tg(CD207-cre)* mice. Spermatozoa which are visible in the testis of *Braf*^*CA*^*Pten*^*loxP/loxP*^ mice are absent from the testis of *Braf*^*CA*^*Pten*^*loxP/loxP*^*Tg(CD207-cre)* mice.

Most of the histological abnormalities present in *Braf*^*CA*^*Tg(CD207-cre)* mice were also seen in *Braf*^*CA*^*Pten*^*loxP/loxP*^*Tg(CD207-cre)* mice. Thymi showed a lack of a sharp cortical-medullary border and a decrease in cortical lymphocytes (Fig 12). Despite some preservation in organ size, spleens again showed loss of well-defined zones of red and white pulp, and testes showed loss of Leydig cells and an absence of spermatic maturation (Fig 12).

**Fig 13:**
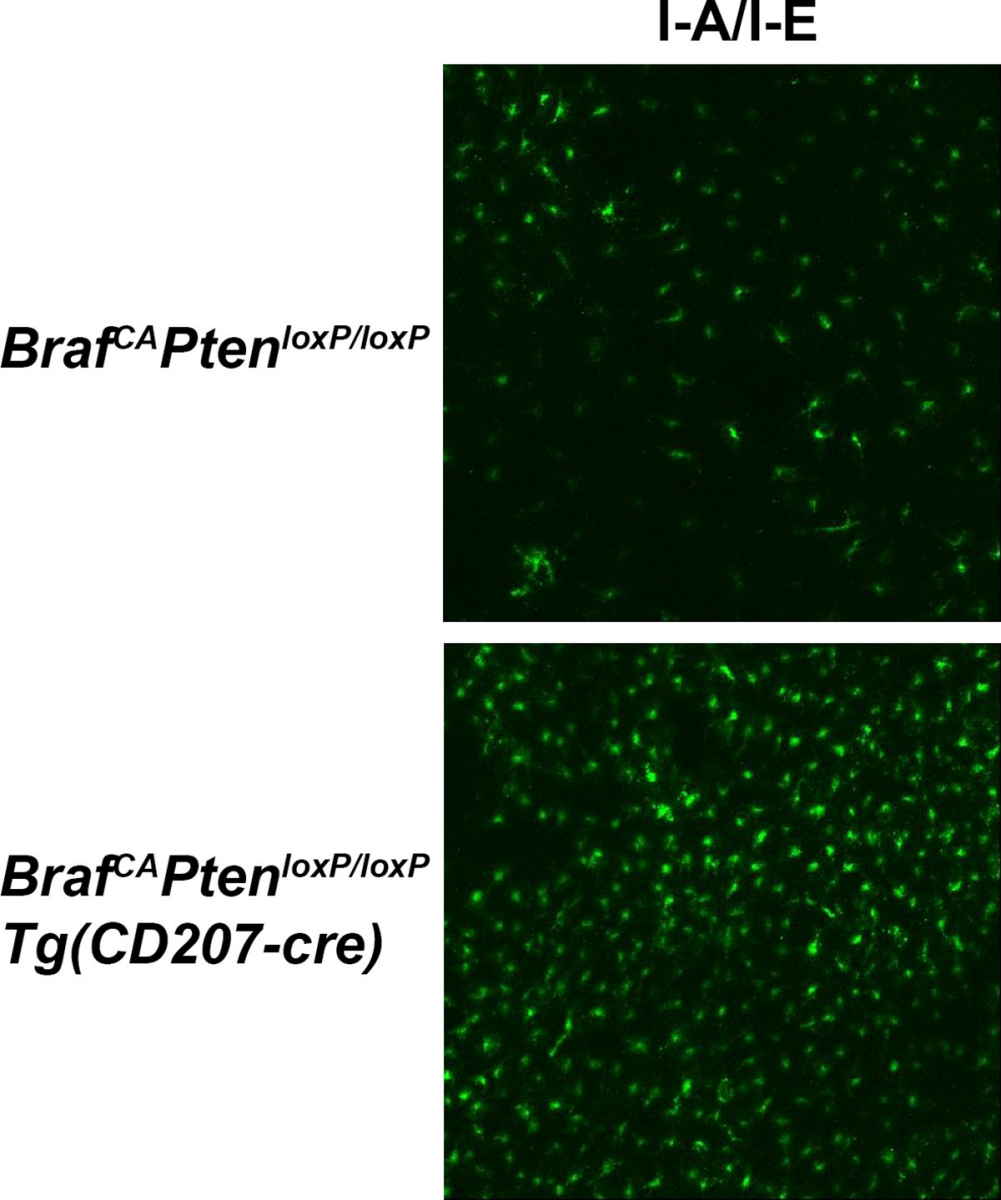
Epidermal Langerhans cells in genetically modified mice. Epidermal sheets were prepared from *Braf*^*CA*^*Pten*^*loxP/loxP*^ and *Braf*^*CA*^*Pten*^*loxP/loxP*^*Tg(CD207-cre)* mice at 15 days of age and stained with fluorescence-conjugated antibodies against MHC Class II (I-A/I-E).

Compared to *Braf*^*CA*^*Tg(CD207-cre)* mice, *Braf*^*CA*^*Pten*^*loxP/loxP*^*Tg(CD207-cre)* mice had an even higher number of epidermal LCs at early ages, 170/field vs. 101/field (p < 0.001 using the mixed model described in Fig 7A) (Fig 7B, Fig 13). That number decreased at 22–28 days of age but, unlike *Braf*^*CA*^*Tg(CD207-cre)* mice, the number of LCs in *Braf*^*CA*^*Pten*^*loxP/loxP*^*Tg(CD207-cre)* remained stable thereafter at a level comparable to control mice and higher than *Braf*^*CA*^*Tg(CD207-cre)* mice. Again, the rearranged *Braf*^*VE*^ allele was present in these LCs (Fig 9).

**Fig 14:**
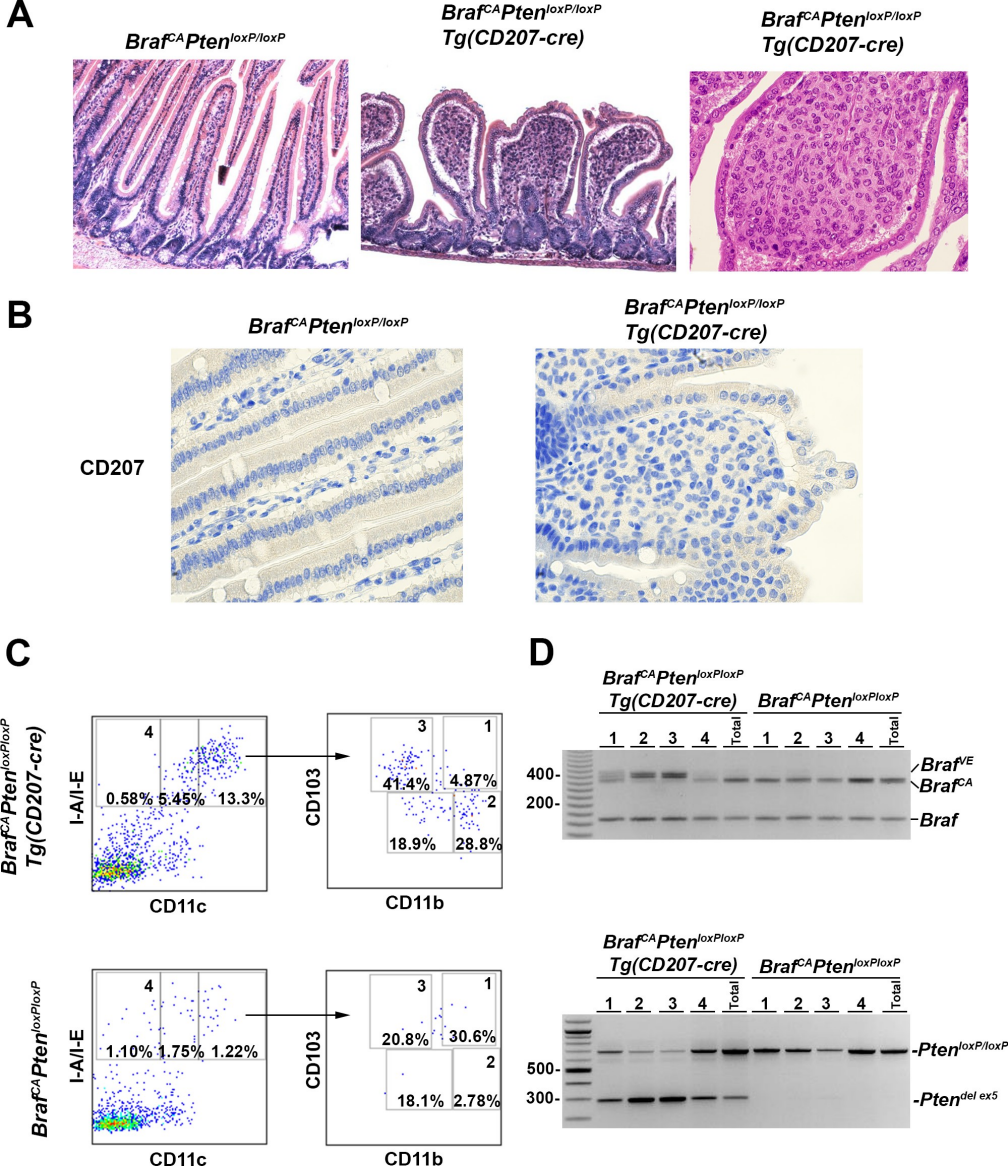
Accumulation of CD103^+^/CD11b^int^ and CD103^int^/CD11b^+^ cells in the small intestine of *Braf*^*CA*^*Pten*^*loxP/loxP*^*Tg(CD207)* mice. **A.** Hematoxylin and eosin staining of representative sections from the small intestine of *Braf*^*CA*^*Pten*^*loxP/loxP*^ (left) and *Braf*^*CA*^*Pten*^*loxP/loxP*^*Tg(CD207-cre)* (middle) for 61 day old mice showing cellular accumulation distorting the villi in the latter. Original magnification, 100X (left and middle). Right, higher power view of infiltrating cells demonstrating their round-to-oval pleiomorphic nuclei and abundant eosinophilic cytoplasm in 29 day old mouse. Original magnification, 400X. **B.** CD207 immunohistochemical staining of the small intestine from *Braf*^*CA*^*Pten*^*loxP/loxP*^ (left) and *Braf*^*CA*^*Pten*^*loxP/loxP*^*Tg(CD207-cre)* (right) 31 day old female mice. No CD207 expression could be detected (see Fig 15A for examples of positive staining). **C.** Cells from the small intestines of *Braf*^*CA*^*Pten*^*loxP/loxP*^*Tg(CD207)* and *Braf*^*CA*^*Pten*^*loxP/loxP*^ 29 day old mice were sorted for I-A/I-E and CD11c. Double positive cells were sorted again for CD103 and CD11b. **D.** DNA was extracted from cells from the indicated regions of the dot plots in **C** and analyzed by PCR for the presence of rearranged *Braf*^*VE*^ and *Pten*^*del ex5*^ alleles. Both rearranged alleles were detected primarily in the CD103^+^/CD11b^int^ and CD103^int^/CD11b^+^ cell fractions with lower amounts in the CD103^+^/CD11b^+^ fraction only from mice that carried the *CD207-cre* allele.

*Dendritic cell accumulation in the small intestine of Braf*^*CA*^*Pten*^*loxP/loxP*^*Tg(CD207-cre) mice*. Surviving *Braf*^*CA*^*Pten*^*loxP/loxP*^*Tg(CD207-cre)* mice displayed a striking phenotype involving accumulation of cells in the lamina propria of the small intestine which distorted the villi (Fig 14A). These cells have the appearance of histiocytes with round-to-oval pleiomorphic nuclei and abundant pale eosinophilic cytoplasm. However, they do not express CD207 by immunohistochemistry (Fig 14B).

To characterize this cell population further, intestinal cells from *Braf*^*CA*^*Pten*^*loxP/loxP*^*Tg(CD207-cre)* and *Braf*^*CA*^*Pten*^*loxP/loxP*^ controls were sorted for I-A/I-E and CD11c expression. Double positive cells were 10-fold more abundant in *Braf*^*CA*^*Pten*^*loxP/loxP*^*Tg(CD207-cre)* mice compared to control *Braf*^*CA*^*Pten*^*loxP/loxP*^ mice (Fig 14C). The majority of these cells were CD103^+^/CD11b^int^ or CD103^int^/CD11b^+^ (Fig 14C), and carried cre-mediated rearrangements of both the *Braf*^*CA*^ and *Pten* alleles (Fig 14D). No such rearrangement was observed in CD11c^-^ cells from the intestines of *Braf*^*CA*^*Pten*^*loxP/loxP*^*Tg(CD207-cre)* or *Braf*^*CA*^*Pten*^*loxP/loxP*^ mice.

### Accumulation of CD207-expressing histiocytes in spleen and lymph nodes of *Braf*^*CA*^*Pten*^*loxP/loxP*^*Tg(CD207-cre)* mice

**Fig 15:**
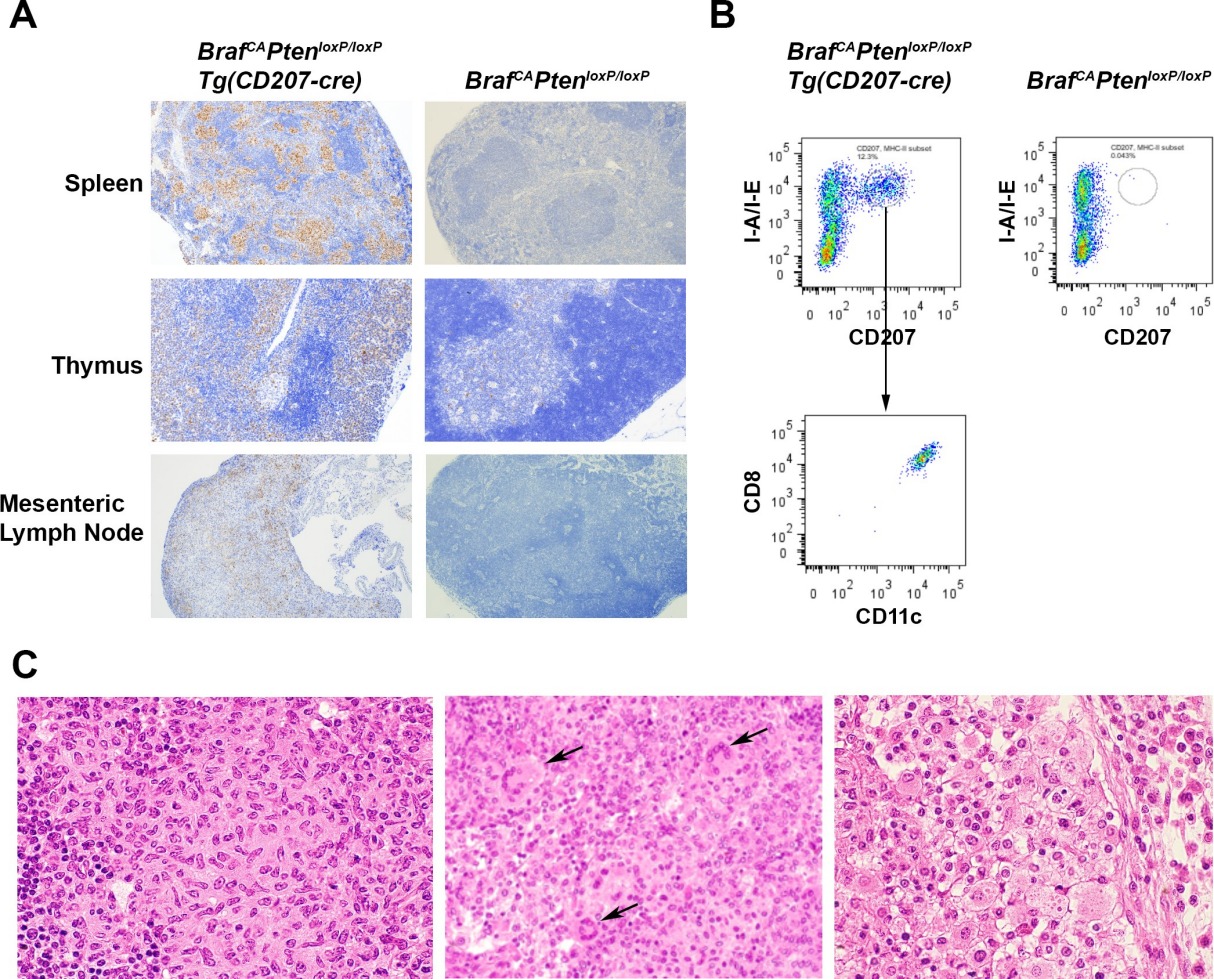
CD207^+^ histiocytes infiltrating the spleen, thymus, and lymph nodes of *Braf*^*CA*^*Pten*^*loxP/loxP*^*Tg(CD207-cre)* mice. **A.** Representative tissue sections from the spleen, thymus, and mesenteric lymph nodes of 36 day old *Braf*^*CA*^*Pten*^*loxP/loxP*^*Tg(CD207-cre)* and *Braf*^*CA*^*Pten*^*loxP/loxP*^ mice were analyzed for CD207-expressing cells by immunohistochemistry. CD207^+^ cells are seen infiltrating all three organs from *Braf*^*CA*^*Pten*^*loxP/loxP*^*Tg(CD207-cre)* but not *Braf*^*CA*^*Pten*^*loxP/loxP*^ mice. Original magnification, 40X. **B.** Spleen cells were sorted for I-A/I-E and CD207 expression. Double positive cells were present in the spleens of 28 day old *Braf*^*CA*^*Pten*^*loxP/loxP*^*Tg(CD207-cre)* but not *Braf*^*CA*^*Pten*^*loxP/loxP*^*Tg(CD207-cre)* mice. These cells were then analyzed for CD8 and CD11c expression and were found to co-express both markers. **C.** Hematoxylin and eosin staining of three regions of a mesenteric lymph node from a female *Braf*^*CA*^*Pten*^*loxP/loxP*^*Tg(CD207-cre)* 32 day old mouse showing histiocytic infiltration. **Left,** histiocytic cells showing folded nuclei and abundant eosinophilic cytoplasm similar to those seen in human LCH cell; **middle,** region showing a similar histiocytic infiltrate but accompanied by multinucleated giant cells (arrows); **right,** region showing a histiocytic infiltrate consisting predominantly of foamy histiocytes similar to those seen in Erdheim-Chester disease. Original magnifications, 100X.

**Fig 16:**
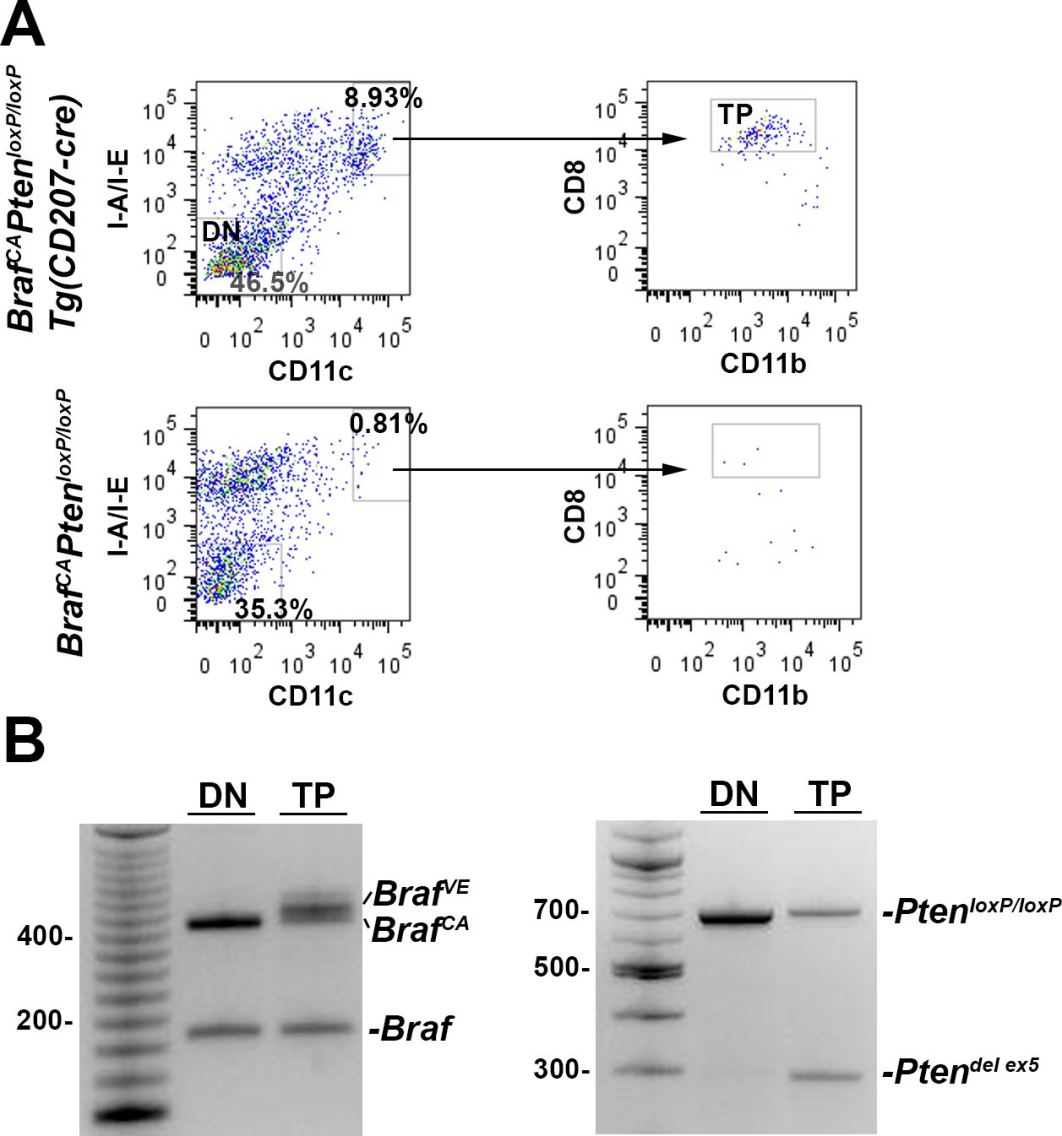
Rearranged *Braf* and *Pten*^*loxP/loxP*^ alleles in the I-A/I-E^+^/CD11c^+^/CD8^+^ cells from the spleens of *Braf*^*CA*^*Pten*^*loxP/loxP*^*Tg(CD207-cre)* mice. **A.** Cells from the spleens of 37 day old *Braf*^*CA*^*Pten*^*loxP/loxP*^*Tg(CD207-cre)* and *Braf*^*CA*^*Pten*^*loxP/loxP*^ mice were sorted for I-A/I-E and CD11c expression. Double Negative (DN) cells are indicated for the *Braf*^*CA*^*Pten*^*loxP/loxP*^*Tg(CD207-cre)* cells. The double positive cells were then sorted for CD8 and CD11b expression. The CD8^+^ cells from *Braf*^*CA*^*Pten*^*loxP/loxP*^*Tg(CD207-cre)* spleens are designated as Triple Positive (TP). **B.** Double Negative (DN) and Triple Positive (TP) cells from **A** were analyzed by PCR for the presence of *Braf* and *Pten* alleles. Cre-mediated rearrangement both alleles was observed in the TP, but not the DN cells.

**Fig 17:**
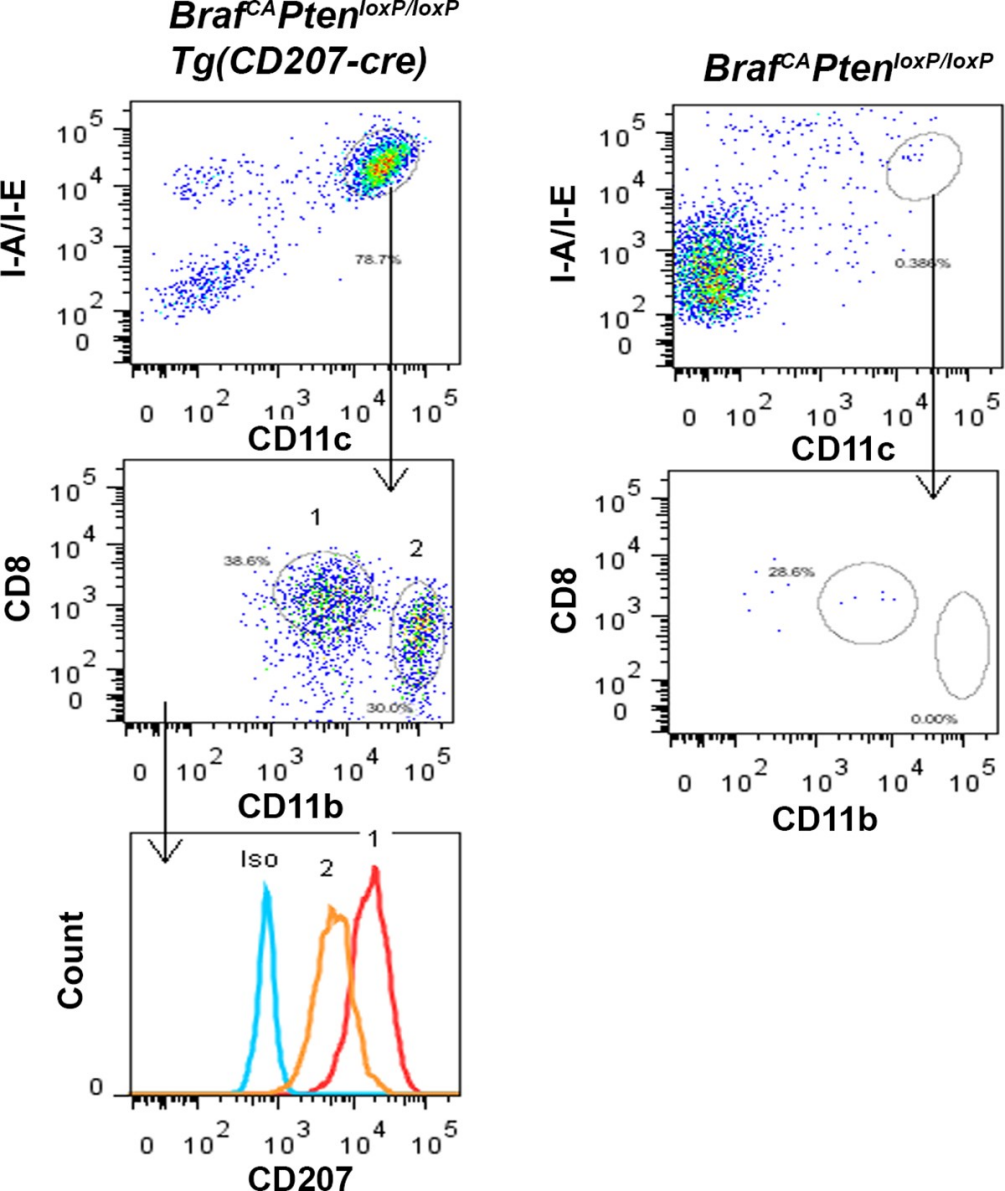
CD207^+^/CD8^-^ cells in the thymus of *Braf*^*CA*^*Pten*^*loxP/loxP*^*Tg(CD207-cre)* mice. Thymic cells from 31 day old *Braf*^*CA*^*Pten*^*loxP/loxP*^*Tg(CD207-cre)* and *Braf*^*CA*^*Pten*^*loxP/loxP*^ mice were sorted for I-A/I-E and CD11c expression. Abundant double positive cells were present in the thymus of *Braf*^*CA*^*Pten*^*loxP/loxP*^*Tg(CD207-cre)* but not *Braf*^*CA*^*Pten*^*loxP/loxP*^ mice. These cells were then analyzed for CD8 and CD11b expression and were resolved into two populations labeled “1” and “2.” Both populations expressed low levels of CD8 compared to spleen cells in Fig 6, and a significant portion of the cells in population 2 were CD8^-^. However, both population 1 and population 2 expressed CD207.

As shown in Fig 11, spleen weight was somewhat preserved in *Braf*^*CA*^*Pten*^*loxP/loxP*^*Tg(CD207-cre)* compared to *Braf*^*CA*^*Tg(CD207-cre)* mice (compare Fig 4). This was due, in part, to the accumulation of CD207^+^ histiocytes in the former (Fig 15A and 15B). Similar cells were also present in the thymus and mesenteric lymph nodes of *Braf*^*CA*^*Pten*^*loxP/loxP*^*Tg(CD207-cre)* mice (Fig 15A). At high power, the morphological features of these cells–coffee bean-shaped nuclei and abundant pale eosinophilic cytoplasm–were reminiscent of human LCH histiocytes (Fig 15C). Multinucleated giant cells, another characteristic of some LCH lesions, were present in mesenteric lymph nodes (Fig 15C). Some fields showed abundant foamy histiocytes similar to those seen in Erdheim-Chester disease (Fig 15C) suggesting a mixed LCH/ECD picture which is increasingly recognized in LCH [19]. However, flow cytometric analysis of the CD207^+^ cells infiltrating the spleen showed that they were CD8^+^ which is not characteristic of LCH histiocytes (Fig 15B). The I-A/I-E^+^/CD11c^+^/CD8^+^ cells in the spleens of *Braf*^*CA*^*Pten*^*loxP/loxP*^*Tg(CD207-cre)* mice, which were absent from the spleens of *Braf*^*CA*^*Pten*^*loxP/loxP*^ mice showed cre-mediated rearrangement of both the *Braf*^*CA*^ and *Pten* alleles (Fig 16). However, a considerable number of I-A/I-E^+^/CD11c^+^/CD8^-^ cells in the thymus were CD207^+^ (Fig 17) which is more characteristic of LCH cells.

## Discussion

We have found that constitutive expression of BRAF V600E driven by the human Langerin promoter in transgenic mice results in significant perinatal mortality. Mice that survive past 21 days of age have a severe phenotype characterized by low weight, reduced thymic and splenic mass, lympho- and monocytopenia, absent Leydig cells with no spermatic maturation, and no survival beyond 65 days of age. Although they have a slight excess of cutaneous LCs at 3 weeks of age, LC number decreases thereafter so that, by 5–6 weeks of age, BRAF V600E-expressing mice have fewer LCs than their control littermates. These mice do not develop LCH-like infiltrates in any organs despite persistence of the rearranged *Braf*^*VE*^ allele in their LCs.

These results are somewhat surprising given the clinical evidence that LCH is a disease caused by single genetic drivers. Patients with a somatic mutation that activates the MAP kinase pathway rarely have concurrent alterations in genes that drive other pathways[2–5, 20] and a growing literature indicates that patients with mutations in MAP kinase pathway genes respond well to targeted therapies.[1, 6–8] Furthermore, Berres et al. demonstrated that transgenic BRAF V600E driven by the murine *Langerin* promoter produces an accumulation of LCH-like cells in a variety of organs reminiscent of a mild form of LCH.[13] One possible explanation for the difference between their model and ours is that the histiocytic accumulation in their mice appears at 12 weeks of age while the *Braf*^*CA*^*Tg(CD207-cre)* mice reported here do not survive beyond 10 weeks, making it impossible to observe such accumulations at later times. However, a more likely explanation is that there is a species difference in both the cell type and timing of Langerin expression. In terms of cell type, both murine and human LCs express Langerin and promoters from both species are active in murine LCs;[21, 22] murine dermal DCs express Langerin[23, 24] but most human dermal DCs do not[25–27], and the human *Langerin* promoter is not active in murine dermal DCs;[23] lamina propria (LP) DCs from C57Bl/6 mice do not express Langerin in the steady state[28] but human LP DCs do,[29–31] and the human *Langerin* promoter is active in murine LP cells.[31] In terms of timing, Langerin is not expressed by murine LCs until day 2 or 3 after birth[32, 33] while Langerin is expressed at 13 weeks gestation by human LC precursors.[34] Our observations (Fig 8) are consistent with this temporal difference in murine and human Langerin expression. Recent data indicate that LCH arises when somatic mutations activating the MAP kinase pathway occur in hematopoietic stem cells.[13] Species specific activities of *Langerin* promoters have not been examined in these precursors but it may be the case that the murine promoter is active in an LCH precursor while the human promoter is not.

The basis for the complex phenotype of *Braf*^*CA*^*Tg(CD207-cre)* mice is not clear but could be interpreted in the context of the senescence-inducing effects of a strong oncogene in normal cells.[9] For example, the gradual reduction in the number of cutaneous LCs might be consistent with the effect of BRAF V600E expression in normal LCs. Components of the phenotype that involve organ atrophy might be due to a similar mechanism if the cells that express the human *Langerin* promoter are involved in organ growth or homeostasis and if their number is diminished by expression of BRAF V600E. For example, testicular macrophage populations are essential for Leydig cell maintenance and testosterone production,[35–37] and it is possible that loss of a testicular macrophage precursor in *Braf*^*CA*^*Tg(CD207-cre)* mice contributed to the absence of Leydig cells and spermatic maturation. Analysis of the ontogeny of testicular macrophage subsets has shown that interstitial macrophages, which are CX_3_CR1^+^CD64^hi^MHCII^-^, are derived from the embryonic yolk sac.[38] Activity of the human *Langerin* promoter in these precursors might explain Leydig cell absence through oncogene-mediated senescence of this subset of testicular macrophages although that remains to be demonstrated. Prenatal activity of the transgenic human Langerin promoter is also consistent with this idea. Loss of Leydig cells could then contribute to the lower levels of testosterone seen in *Braf*^*CA*^*Tg(CD207-cre)* mice which may, in turn, contribute to the smaller size of these mice.[39–41] The fact that testosterone levels were only reduced by 46% compared to control mice suggests the possibility of alternative sources of androgen such as adrenal gland synthesis of DHEA or other precursors which could be converted to testosterone in the periphery. [42] However, androgen deprivation alone cannot account for their early deaths and the decreased size of their thymi and spleens.[40, 41]

One problem with this mechanism is the extraordinarily mild phenotype of mice in which cells that activate the human *Langerin* promoter are ablated via expression of diphtheria toxin fragment A.[21] They do not phenocopy *Braf*^*CA*^*Tg(CD207-cre)* mice. The major difference in the two models is that one involves toxin-induced ablation of a cell lineage and the other involves the expression of a strong oncogene. This suggests that expression of BRAF V600E in relevant cells may produce effects beyond those of oncogene-induced senescence in order to create the phenotype observed in *Braf*^*CA*^*Tg(CD207-cre)* mice.

Nonetheless, if oncogene-induced senescence is at least partly responsible for the absence of an LCH-like phenotype in *Braf*^*CA*^*Tg(CD207-cre)* mice, this could be tested by interrupting the cellular pathways that induce this state. For example, BRAF V600E-induced senescence in melanocytes can be overcome by deleting *PTEN*.[10] This was our rationale for developing *Braf*^*CA*^*Pten*^*loxP/loxP*^*Tg(CD207-cre)* mice. However, the phenotype of *Braf*^*CA*^*Pten*^*loxP/loxP*^*Tg(CD207-cre)* mice was complex and unexpected. Their shortened survival, lympho- and monocytopenia, absence of Leydig cells, and thymic and splenic atrophy were nearly identical to *Braf*^*CA*^*Tg(CD207-cre)* mice. These observations indicate that *Pten* disruption did not rescue these components of the *Braf*^*CA*^*Tg(CD207-cre)* phenotype.

However, *Braf*^*CA*^*Pten*^*loxP/loxP*^*Tg(CD207-cre)* mice had novel proliferative phenotypes. At 2–3 weeks of age, the number of cutaneous LCs in *Braf*^*CA*^*Pten*^*loxP/loxP*^*Tg(CD207-cre)* mice was 68% higher than the number in *Braf*^*CA*^*Tg(CD207-cre)* mice. If the proliferative stimulus provided by BRAF V600E were counterbalanced by tumor suppressor-mediated senescence, this result might indicate that the drive toward senescence was at least partially blocked by disrupting *Pten*. Still, at 4–5 weeks, the number of LCs declined to the level seen in control mice. Thereafter, however, the number remained stable rather than decreasing still further as seen in *Braf*^*CA*^*Tg(CD207-cre)* mice, suggesting that *Pten* disruption may have continued to be effective.

*Braf*^*CA*^*Pten*^*loxP/loxP*^*Tg(CD207-cre)* mice also displayed two additional phenotypes. The first was a 10-fold expansion of MHCII^+^CD11c^+^ cells in the small intestine compared to controls and to *Braf*^*CA*^*Tg(CD207-cre)* mice. Nearly all of these cells expressed CD103 and CD11b. Molecular analysis showed that these cells harbor rearranged *Braf*^*VE*^ and *Pten*^*del ex5*^ indicating that their accumulation is likely a cell-autonomous effect. Species-specific patterns of Langerin expression are also consistent with this model. Under steady state conditions, murine CD103^+^CD11b^+^ cells in the LP do not express Langerin[28] but human LP DCs do[29, 30] and, as noted above, the human *Langerin* promoter is active in murine LP CD103^+^CD11b^+^ cells.[31] Clearly, expression of BRAF V600E alone is insufficient to lead to the expansion of LP CD103^+^CD11b^+^ cells since this was not observed in *Braf*^*CA*^*Tg(CD207-cre)* mice. However, disruption of *Pten* created a permissive context which allowed these cells to proliferate. Could this be a model of gastrointestinal LCH? This is unlikely because gastrointestinal LCH cells can be CD207^+^ while the LP histiocytes that accumulate in *Braf*^*CA*^*Pten*^*loxP/loxP*^*Tg(CD207-cre)* mice are CD207^-^.

The second striking phenotype seen in these mice is somewhat more reminiscent of LCH. *Braf*^*CA*^*Pten*^*loxP/loxP*^*Tg(CD207-cre)* mice showed an accumulation of CD207^+^ cells in the spleen, thymus, and lymph nodes. The lesions were filled with histiocytes having a morphology similar to LCH cells and were accompanied by multinucleated giant cells, a common feature of LCH. Furthermore, some regions contained foamy histiocytes similar to those seen in Erdheim-Chester disease (ECD). Together, these observations suggest the possibility that *Braf*^*CA*^*Pten*^*loxP/loxP*^*Tg(CD207-cre)* mice could be a model of multisystem mixed LCH/ECD. The frequency of mixed disease in one ECD cohort is 19% [19] and BRAF V600E is a common driver in both diseases.[43] The discovery of concurrent *PIK3CA* and *BRAF* mutations in a proportion of ECD patients may be particularly relevant to our *Pten*-disrupted model.[44] However, most of the histocytes that accumulate in the spleen and lymph nodes of these mice are CD8^+^, unlike LCH cells. This may be a consequence of the fact that, in contrast to humans, murine resident CD207^+^ cells in spleen and lymph nodes are CD8^+^ [45]. In contrast, the histiocytes that accumulate in the thymus of *Braf*^*CA*^*Pten*^*loxP/loxP*^*Tg(CD207-cre)* mice are CD8^-^ and more closely approximate the phenotype of LCH cells.

We chose to bypass senescence signaling by disrupting *Pten*, in part, because of the role it plays in permitting BRAF V600E-driven melanocyte proliferation. But we also chose it because, when these experiments were being planned, a case report had appeared which described a major clinical response to an AKT inhibitor in an LCH patient.[46] However, a clinical trial of the inhibitor in LCH patients showed minimal activity[47] and further genetic analysis of a large number of LCH cases showed only rare disruption or alteration of the PTEN/AKT/PIK3C pathway.[4, 5, 48] In fact, very few LCH cases with alterations in MAP kinase pathway genes show genetic alterations affecting other pathways. Nonetheless, for reasons not yet understood, TP53 is almost always overexpressed in LCH cells suggesting some kind of dysregulation of this tumor suppressor.[49] Models in which disrupted or hypomorphic *TP53* alleles are combined with BRAF V600E expression might be more likely to mimic LCH.

## Supporting information

S1 NC3Rs ARRIVE Guidelines Checklist ARRIVE (Animal Research: Reporting of *In Vivo* Experiments) checklist from the National Centre for the Replacement, Refinement, & Reduction of Animals in Research shows where in this manuscript the recommendations can be found. (PDF)
